# Primary cesarean section in Sub-Saharan Africa: A systematic review and meta-analysis using the Robson Ten-Group Classification System

**DOI:** 10.1371/journal.pone.0354911

**Published:** 2026-07-30

**Authors:** Kindu Yinges Wondie, Endalk Birrie Wondifraw, Miteku Andualem Limenih, Berihun Agegn Mengistie, Getie Mihret Aragaw, Nuhamin Tesfa Tsega, Zelalem Nigussie Azene

**Affiliations:** 1 Department of Clinical Midwifery, School of Midwifery, College of Medicine and Health Sciences, University of Gondar, Gondar, Ethiopia; 2 Department of Child Health Nursing, College of Medicine and Health Sciences, Wollo University, Dessie, Ethiopia; 3 Department of General Midwifery, School of Midwifery, College of Medicine and Health Sciences, University of Gondar, Gondar, Ethiopia; 4 Department of Women’s and Family Health, School of Midwifery, College of Medicine and Health Sciences, University of Gondar, Gondar, Ethiopia; FMUP: Universidade do Porto Faculdade de Medicina, PORTUGAL

## Abstract

**Background:**

The overuse of cesarean sections, mainly of primary procedures, is a major contributor to perinatal mortality in Sub-Saharan Africa. The Robson Ten-Group Classification System is the global standard for cesarean section rate. Evidence on the pooled primary cesarean section rate using this system in Sub-Saharan Africa SSA remains limited.

**Materials and methods:**

A systematic review and meta-analysis were conducted. PubMed, Scopus, ScienceDirect, Google Scholar, Google, and ResearchGate were searched for studies published between 2001 and February 22, 2025. Data were extracted into Microsoft Excel and analyzed using Stata 17. A random-effects model was applied to calculate the pooled rate. Study quality was assessed using the Joanna Briggs Institute Critical Appraisal Checklist for Studies Reporting Prevalence Data. Publication bias was assessed using a Doi plot and the Luis Furuya-Kanamori index. Heterogeneity was assessed using the I² statistic.

**Results:**

The pooled facility‑based primary cesarean section rate, derived from 25 institution‑based studies (213,117 births; predominantly secondary and tertiary hospitals), in Sub-Saharan Africa was 18.7% (95% CI: 16.2–21.4; 95% prediction interval: 8.4%−36.8%). Robson Groups 1 and 2 were the largest contributors (47.5%), with Group 1 alone accounting for 37.1%. In both nulliparous and multiparous women, primary cesarean section was more common after spontaneous labor than after induction or elective procedures. Significant heterogeneity was observed across studies (I² = 99.64%, p < 0.001). No evidence of publication bias was found (LFK index = −0.27).

**Conclusion:**

The facility‑based primary cesarean section rate in Sub-Saharan Africa exceeded the WHO global reference, with Robson Groups 1 and 2 being the largest contributors. High rates of prelabor cesarean and elevated rates following spontaneous labor raise concern for possible overuse, but unmeasured comorbidities and referral bias cannot be excluded. Stricter adherence to evidence-based protocols, routine use of the Robson classification for clinical audits, and linkage with indication‑level data are essential for improving obstetric care.

## Introduction

Cesarean section (CS) is a key indicator of access to safe and timely obstetric care [1]. Globally, CS rates are projected to reach nearly 30% by 2030, with 90% of procedures occurring in resource-limited settings [2]. In Sub-Saharan Africa (SSA), the overall rate remains the lowest worldwide at 7.1% [2]. This paradox, underuse among high-risk women and overuse among low-risk women, drives preventable maternal and neonatal mortality and morbidity [2–6].

Primary CS (PCS), defined as a woman’s first CS, is the main driver of overall CS rates and initiates a cascade of repeat procedures in future pregnancies [7]. PCS is frequently overused in low-risk women for whom vaginal birth is feasible [8], associated with increasing risk of short- and long-term complications [9]. In low-resource, high-fertility settings such as SSA, unnecessary PCS further escalates overall CS rates and associated risks [6]. Reducing unnecessary PCS is therefore a global maternal health priority; monitoring PCS rates is essential to optimize resource allocation, address disparities, and monitor alignment with World Health Organization (WHO) recommendations [10,11].

The Robson Ten-Group Classification System is the gold standard for monitoring and reducing unnecessary CS [12]. It classifies all women into one of 10 mutually exclusive groups based on parity, previous CS, fetal number and presentation, gestational age, and onset of labor [13]. Although standardized PCS assessment is a global priority [14], evidence from SSA remains scarce and largely limited to single-facility studies. This systematic review and meta-analysis therefore aimed to estimate the pooled PCS rate in SSA using the Robson classification and to identify the major contributing obstetric groups [15].

## Materials and methods

### Protocol and guidelines

This systematic review and meta-analysis followed the Preferred Reporting Items for Systematic Reviews and Meta-Analyses (PRISMA) guidelines [16] (S1 File). The protocol was prospectively registered with the International Prospective Register of Systematic Reviews (CRD42024623227).

### Search strategy

We searched Scopus, PubMed, ScienceDirect, Google Scholar, ResearchGate, and Google up to February 22, 2025, using the CoCoPop framework (condition, context, population). Free-text terms were combined with Boolean operators (AND, OR, NOT). Search terms included “Africa,” “Sub-Saharan Africa,” and individual SSA country names. Four reviewers (KYW, EBW, MAL, ZNA) independently performed the searches (S2 File).

### Eligibility criteria

#### Inclusion criteria.

Observational studies (cross-sectional, cohort, or case-control) conducted in SSA that used the WHO Robson Ten-Group Classification System and reported fully disaggregated data for Robson Groups 1–10 were eligible, with no date restriction.

#### Exclusion criteria.

Studies were excluded if they did not report complete Robson group data, used aggregated/incomplete/inconsistent classifications, lacked sufficient data for PCS estimation, or were reviews, qualitative studies, case reports, editorials, abstracts, or methodological papers.

### Screening and selection of studies

Records were imported into EndNote 21 and duplicates removed. Titles and abstracts were screened independently by three reviewers (KYW, BAM, GMA), followed by full-text review. Disagreements were resolved by discussion and consensus; if consensus was not reached, a third reviewer (senior author) made the final decision.

### Outcome measurement

The outcome was the proportion of primary cesarean section (PCS). PCS was operationally defined by the review team using the Robson Ten-Group Classification System as cesarean deliveries occurring in women without a previous cesarean section, corresponding to Robson Groups 1, 2, 3, 4, and 6 [13].

Robson Group 5 (women with at least one previous cesarean section) was excluded by definition. Although Groups 7–10 are not defined by previous cesarean status, they may include a mixture of women with and without prior cesarean section and represent heterogeneous and often higher-risk obstetric conditions (e.g., breech presentation, multiple pregnancy, abnormal lie, and preterm birth) [13]. Therefore, these groups were excluded from the PCS definition to ensure conceptual clarity and comparability across studies. The prevalence of PCS was calculated as:


$$\begin{array}{c} \text{PCS prevalence}=\frac{(\sum \text{number of cesarean births in Robson Groups }\text{1},\;\text{2},\;\text{3},\;\text{4},\text{ and }\text{6})\;}{\left(\sum \text{total number of women in all Robson Groups }\text{1}-\text{10}\right)} \end{array}$$

Group-specific cesarean section (CS) prevalence was calculated as the number of cesarean deliveries within each Robson group divided by the total number of women in that group.

Where reported, Robson subcategories (e.g., 2A/2B and 4A/4B) were analyzed separately to examine differences by onset of labor (spontaneous, induced, or prelabor).

### Robson group classification and data harmonization

Robson group data were extracted as reported in the original studies without reclassification. To ensure methodological consistency, only studies that explicitly applied the standard Robson Ten-Group Classification System [13] and reported all ten groups were included.

For the primary analysis, subcategories (e.g., 2A/2B and 4A/4B) were aggregated into their respective main Robson groups. No reconstruction of Robson groups from individual obstetric variables was performed. Studies with incomplete, inconsistent, or unverifiable Robson classification data were excluded. Because all included studies reported complete Robson group distributions, no imputation of missing data was undertaken.

Where data permitted, additional analyses were conducted to explore cesarean section rates by onset of labor.

### Quality assessment

Study quality was assessed the Joanna Briggs Institute (JBI) Critical Appraisal Checklist for Studies Reporting Prevalence Data, with scores ranging 0–9 [17]. Five authors (KYW, EBW, MAL, BAM, and GMA) independently appraised the methodological quality of the included studies, and any discrepancies were resolved through discussion and consensus. No study was excluded on the basis of quality assessment. JBI appraisal results were used to assess methodological quality and inform the interpretation of findings.

### Data extraction and management

Four reviewers (KYW, BAM, GMA, NTS) independently extracted data using a standardized Excel form. Extracted variables included study characteristics (first author, publication year, data collection year, country, income level, CS cost-exemption policy, SSA subregion, study design, sampling technique, and facility type and level) and methodological quality (JBI score). Quantitative data included total sample size, total births, Robson group sizes (Groups 1–10), cesarean section (CS) counts per group, total primary cesarean section (PCS) cases, and group-specific CS numbers.

The complete extracted dataset, cleaned Stata dataset, and analysis code are publicly available in Zenodo at https://doi.org/10.5281/zenodo.19644772.

Missing or unclear data were requested from corresponding authors by email. Disagreements were resolved through discussion and consensus. Inter-rater agreement was assessed using Cohen’s kappa.

### Data synthesis and statistical analysis

This meta-analysis of prevalence was guided by the PERSyst (Preferred Reporting Items for Systematic reviews and Meta-analyses of prevalence studies) framework [18]. Statistical analyses were performed using Stata version 17.0 (StataCorp, College Station, TX, USA). Study-specific proportions were calculated as the number of primary cesarean sections (PCS) divided by total births. Primary cesarean section rates were treated as proportions and pooled using a random-effects meta-analysis based on the DerSimonian–Laird method to account for anticipated clinical and methodological heterogeneity across studies. To stabilize variances and improve the normality of proportion estimates, logit-transformation was applied before pooling, and pooled estimates were subsequently back-transformed to the original proportion scale for interpretation [18]. Standard errors were derived within the Stata meta-analysis framework using the logit transformation [19]. All analyses were conducted using Stata’s built-in meta commands.

Between-study heterogeneity was assessed using Cochran’s Q test and the I² statistic [20]. Although I² values of 25%, 50%, and 75% are commonly interpreted as representing low, moderate, and high heterogeneity, respectively, very high I² values are frequently observed in meta-analyses of prevalence estimates and may have limited discriminatory value. Therefore, I² was interpreted cautiously, and heterogeneity was further explored through prespecified subgroup analyses and sensitivity analyses rather than relying solely on conventional I² thresholds [21].

Prespecified subgroup analyses were conducted to explore sources of heterogeneity by study period, Sub-Saharan Africa region, country income level, facility type, facility level, cesarean section cost-exemption policy, and study quality.

To further explore potential sources of between-study heterogeneity, univariable random-effects meta-regression analyses were performed using study period, geographic region, country income category, institution type, facility level, and cesarean section cost-exemption policy as study-level covariates.

Sensitivity analyses included leave-one-out analysis, influence diagnostics, and restriction of the meta-analysis to studies of higher methodological quality.

Small-study effects were assessed using Doi plots and the Luis Furuya-Kanamori (LFK) index [22]. An absolute LFK index value <1 was considered indicative of no asymmetry, values between 1 and 2 indicated minor asymmetry, and values >2 indicated major asymmetry [22].

Results are presented using forest plots, tables, and narrative summaries.

## Results

### Study selection

**Fig 1:**
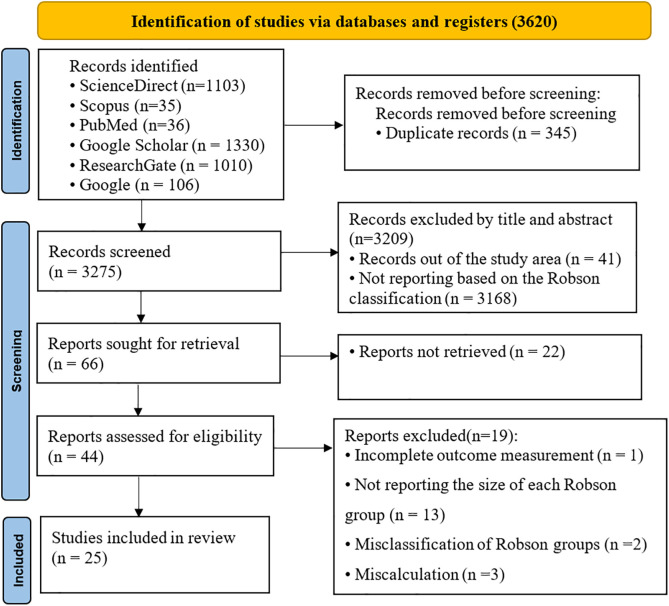
PRISMA flow diagram of study selection.

The database search yielded 3,620 records. After removing 345 duplicates, 3,275 titles and abstracts were screened; 3,209 were excluded and 22 full-text articles were unavailable. Forty-four full-text articles were assessed, of which 19 were excluded (absence of Robson group sizes, n = 13 [23–35]; incorrect CS calculations, n = 3 [36–38]; misclassification, n = 2 [39,40]; incomplete reporting, n = 1) [41]. Twenty-five studies [42–66] were included **(**Fig 1).

### Characteristics of studies included

Twenty-five institution-based cross-sectional studies comprising 213,117 births (66,539 primary cesarean sections) were analyzed.

The methodological quality of the included studies was assessed using the JBI Critical Appraisal Checklist for Studies Reporting Prevalence Data. All included studies scored between 7 and 9 on the JBI checklist, with 21 studies scoring 8–9 and four studies scoring 7 (S3 File). Five studies [43,45,58,63,66] were conducted in 2011–2015, 12 [42,44,48,53–57,61,62,64,65] in 2016–2020, and eight [46,47,49–52,59,60] in 2021–2025. Thirteen studies [46,47,49–52,59,60] were from Eastern Africa and 12 [43,45–47,51,54,56,57,60,62–64] from Western Africa; 13 [42–44,47–51,53,55,57,61,65] were from low-income and 12 [45,46,52,54,56,59,60,62–64,66] from lower-middle-income countries. Five [42,49,54,55,63] used prospective designs and 20 were retrospective. Fourteen [44,45,47,48,52,53,57–59,62,63,66] applied census sampling. Eighteen [42–45,47,49,52,54,55,57–65] were conducted in public facilities, four in private, and three in mixed settings (S4 File).

### Pooled prevalence of primary cesarean section

**Fig 2:**
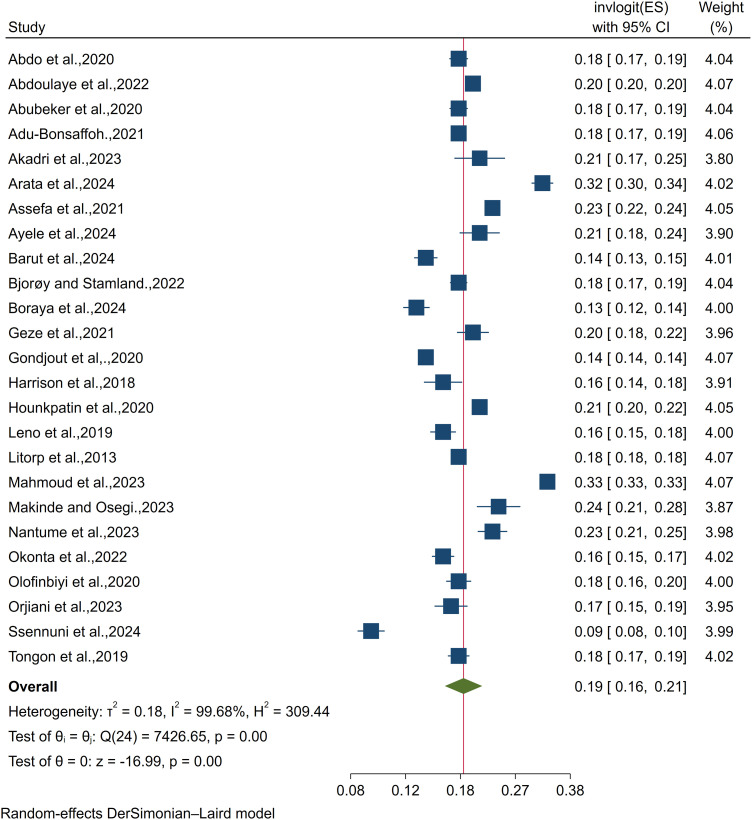
Pooled prevalence of primary cesarean section using a logit-transformed random-effects model.

The random-effects pooled prevalence of primary cesarean section (PCS) based on the included studies was 18.7% (95% CI: 16.2–21.4). The 95% prediction interval ranged from 8.4% to 36.8%, reflecting the substantial between‑study heterogeneity. Heterogeneity was high (I² = 99.68%, p < 0.001) **(**Fig 2).

### Contribution of Robson groups to primary cesarean section

Robson Groups 1–4 accounted for 94.6% of PCS. Groups 1 and 2 (nulliparous, term, singleton, cephalic) represented 47.5% of pregnancies, with Group 1 contributing the largest share (37.1%). Group 3 contributed 34.1%. Nulliparous pregnancies (Groups 1, 2, and 6) accounted for 52.9% of PCS overall. Provider-initiated cesareans (Groups 2 and 4) contributed 14.4% of total PCS (Table 1).

**Table 1:** Size and contribution of the Robson groups to the overall rate of primary cesarean section in SSA.

Groups with no previous CS (1 –6)	Number of women admitted to labor in group (C)	Number of CSs in group (E)	Group CS rate (%) (E/C)	Group absolute contribution deliveries % (E/A)	Group relative contribution deliveries with no previous CS (E/D)	Group relative contribution to CS %(E/B)	Group relative contribution to primary CS (%) (E/F)
1	75983	24697	32.5	7.5	11.6	19.7	37.1
2	18014	6936	38.5	2.1	3.3	5.5	10.4
3	98209	22685	23.1	6.9	10.6	18.1	34.1
4	15250	8611	56.5	2.6	4.0	6.9	12.9
6	5661	3610	63.8	1.1	1.7	2.9	5.4
total	213117(D)	66539(F)	31.233	20.2	31.2	53.2	100

Displays Robson group size, cesarean section rate, absolute contribution to the total number of births across all Robson groups, relative contribution to the total number of births with no previous cesarean section, total number of cesarean sections across all Robson groups, and total number of primary cesarean section births.

A = Total number of women admitted to labor in all Robson groups = 329344

B = Total number of women who underwent cesarean section in all Robson groups = 125070

CS = cesarean section.

### Primary cesarean section according to onset of labor

Ten studies provided detailed onset-of-labor data (20,617 women without prior CS; 5,512 cesareans). The cesarean rate after spontaneous labor (Groups 1 and 3) was 18.8%, compared with 89.8% for prelabor cesareans (Groups 2B and 4B) and 31.8% after induction (Groups 2A and 4A) (Table 2).

**Table 2:** Rate of primary cesarean section based on the type of onset of labor after which the cesarean section was performed among provider-initiated CS in SSA.

Groups with no previous CS (1 –4)	Number of women admitted to labor in group (C)	Number of CSs in group (E)	Group CS rate (%) (E/C)	Group absolute contribution deliveries % (E/A)	Group relative contribution deliveries with no previous CS (E/D)	Group relative contribution to CS %(E/B)	Group relative contribution to primary CS (%) (E/F)
1	7757	1979	25.51	6.9	9.6	19.1	35.9
2	1880	1187	63.14	4.1	5.8	7.6	7.2
2A	1032	399	38.66	1.4	1.9	3.8	7.2
2B	848	788	92.92	2.7	3.8	7.6	14.3
3	9062	1190	13.13	4.1	5.8	11.5	21.6
4	1466	834	56.9	4.0	8.0	15.1	15.1
4A	663	140	21.12	0.5	0.7	1.3	2.5
4B	803	694	86.43	2.4	3.4	6.7	12.6
Induced(2A + 4A)	1695	539	31.80	1.9	2.6	5.2	9.8
Prelabor(2B + 4B)	1651	1482	89.76	5.1	7.2	14.3	26.9
Spontaneous (1 + 3)	16819	3169	18.84	11.0	15.4	30.5	57.5
Total	20165(D)	5190(F)	25.7	18.0	25.2	50.0	100.0

A = Total number of women admitted to labor in all Robson groups 1, 2, 3, and 4 = 28879

B = Total number of women who underwent CS in Robson groups 1, 2, 3, and 4 = 10387

CS = cesarean section.

### Subgroup analysis

No significant differences were found by study period (p = 0.446), region (p = 0.674), country income level (p = 0.908), study quality (p = 0.405), institution level (p = 0.108), or institution type (p = 0.406). A significant difference existed by cesarean cost-exemption policy (p = 0.041), with the highest PCS prevalence in settings with universal/near-universal exemption (19.4%, 95% CI: 16.4–22.9). Heterogeneity remained high within subgroups (Table 3).

**Table 3:** Subgroup meta-analysis of pooled prevalence estimates according to study characteristics and contextual factors among included African studies.

Variable	Subgroup category	Studies (n)	Pooled prevalence, %	95% confidence interval	I² within subgroups (%)	Over all % I²	P-value for subgroup difference
Study period	2011-2015	5	18.4	17.5-19.4	93.73	99.68	0.446
	2016-2020	12	17.2	14.9-19.8	98.19		
	2021-2025	8	21.2	15.3-28.7	99.44		
Region	East Africa	13	18.2	14.3-22.7	99.79	99.68	0.674
	West Africa	12	19.2	17.0-21.6	98.56		
Country income level	Low-income	13	18.5	16.3-21.0	97.9	99.68	0.908
	Lower-middle income	12	18.8	14.9-23.4	99.84		
Study quality (JBI score)	8-9	21	18.4	15.7-21.3	99.73	99.68	0.405
	5-7	4	20.3	16.9-24.1	81.51		
Cesarean section cost policy in study country	Universally exempted	17	19.4	16.4-22.9	99.78	99.68	0.041*
	Partially exempted	6	17.7	15.2-20.6	91.04		
	Not exempted	2	14.9	13.1-17.0	75.9		
Health facility level included in the study	Tertiary only	20	19.1	16.3-22.3	99.69	99.68	0.295
	Multiple facility levels	5	16.9	14.1-20.1	98.07		
Health facility type included in the study	Public	18	18.5	15.5-21.9	99.77	99.68	0.406
	Private	4	17.9	15.0-21.3	91.34		
	Public and private	3	20.6	18.0-23.5	94.94		

* Statistically significant at p < 0.05. I², Higgins’ inconsistency statistic. Random-effects meta-analysis was performed using the DerSimonian–Laird method with inverse logit transformation. Between-group differences were assessed using the Q-test for subgroup heterogeneity. Statistical significance was set at p < 0.05.

**Fig 3:**
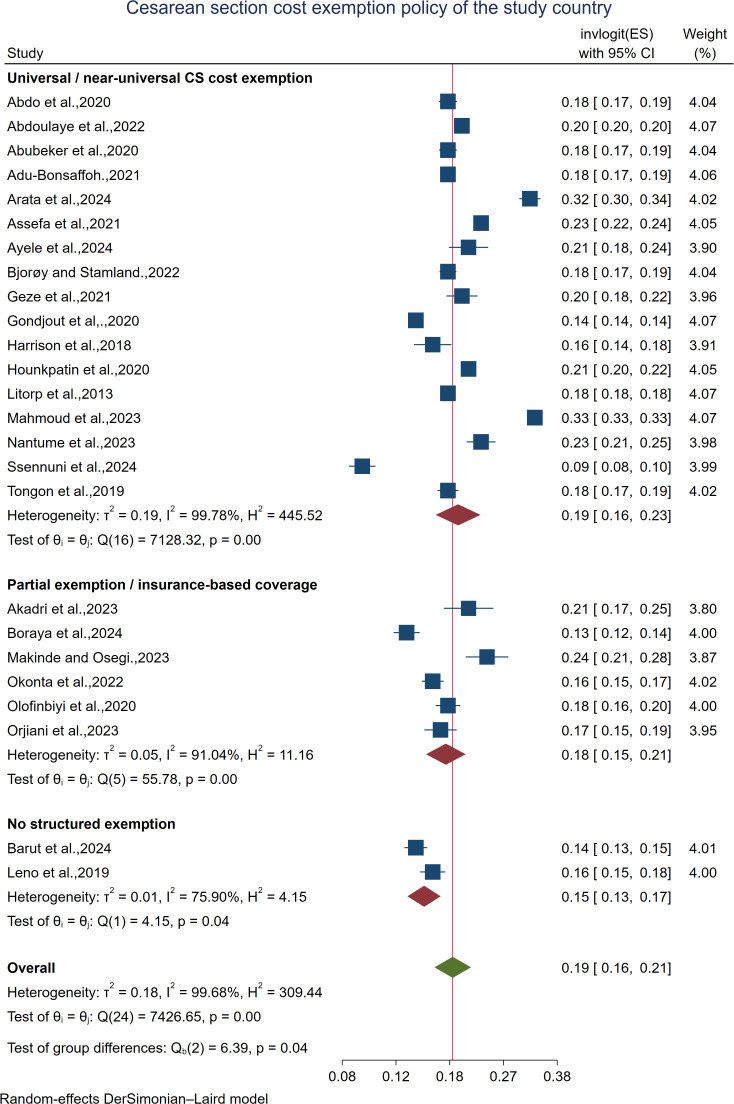
Subgroup analysis of prevalence primary cesarean section in Sub-Saharan Africa based on national cesarean cost exemption policy.

Forest plot for sub-group analysis of cesarean cost exemption policy is given in the figure (Fig 3).

Univariable meta-regression analyses were conducted to assess whether study period, geographic region, country income category, institution type, facility level, and cesarean section cost-exemption policy explained between-study heterogeneity (Table 4). None of the examined study-level characteristics were significantly associated with PCS prevalence (all p > 0.05). Study period accounted for the largest proportion of between-study variance (R² = 56.1%), although the association did not reach statistical significance (p = 0.147).

**Table 4:** Univariable meta-regression of study-level characteristics associated with PCS prevalence.

Covariate	Category	β Coefficient	95% CI	p-value
Study period	2011 - 2015 (Ref)			
	2016 - 2020	−0.08	−0.380 - 0.220	0.601
	2021 - 2025	0.18	−0.143 - 0.503	0.275
Facility level	Tertiary only (Ref)			
	Multiple facilities	−0.153	−0.569 - 0.262	0.469
Geographic region	East Africa (Ref)			
	West Africa	0.07	−0.279 - 0.419	0.692
Country income level	Low-income (Ref)			
	Lower-middle income	0.019	−0.339 - 0.378	0.916
Institution type	Public (ref)			
	Private	−0.03	−0.512 - 0.452	0.903
	Both	0.134	−0.405 - 0.672	0.626

### Publication bias

**Fig 4:**
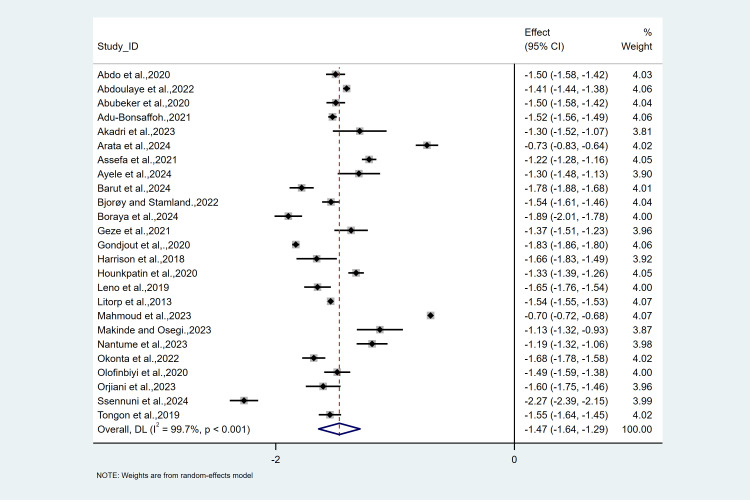
Doi plot for publication bias assessment of primary cesarean section prevalence studies in Sub-Saharan Africa.

The Doi plot (Fig 4) showed no major asymmetry, with an LFK index of −0.27 (within the ± 1 range for no asymmetry), indicating no significant publication bias.

### Sensitivity analysis

**Fig 5:**
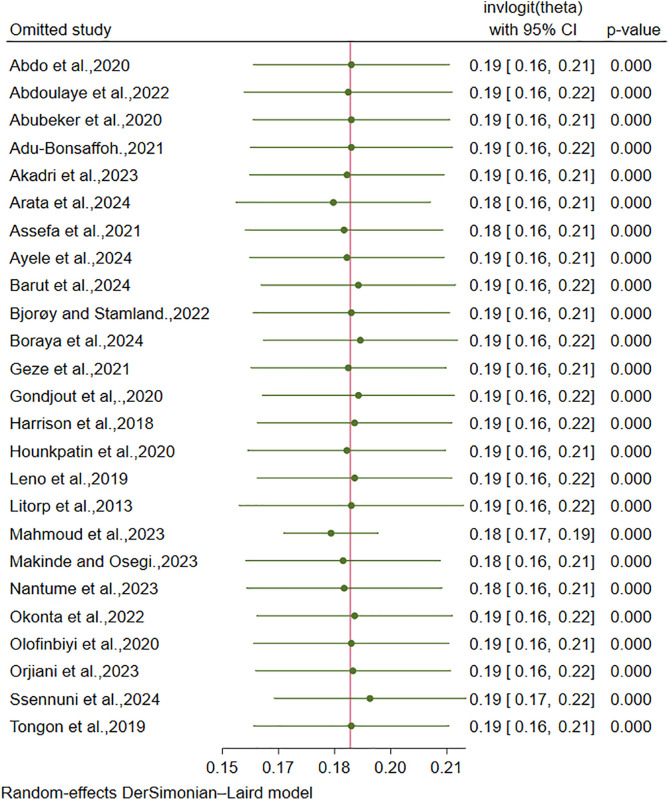
Leave-one-out sensitivity analysis of included studies.

Leave-one-out analysis confirmed stability (pooled estimates 18.1–19.2%) The random-effects model was retained given persistent high heterogeneity (Fig 5).

## Discussion

This systematic review and meta-analysis found a pooled prevalence of primary cesarean section (PCS) of 18.7% (95% CI: 16.2–21.4) among the included studies from Sub-Saharan Africa (SSA), all of which were institution‑based (predominantly secondary and tertiary hospitals). This rate exceeds the WHO global reference of 11.7% [67]. However, because the estimate reflects facility‑level practice in referral settings, it should not be interpreted as the population‑level PCS rate for all births in SSA. This elevated facility‑based rate reflects the complex interplay of a high burden of obstetric complications, strained healthcare systems, and variable clinical decision-making across the region [68].

Although the high heterogeneity (I² = 99.68%) limits the interpretability of a single pooled estimate, we elected to pool studies because the Robson classification provides a standardized framework that enables meaningful comparison across diverse SSA settings. The random‑effects model accounts for between‑study variance, and the pooled estimate should be interpreted as a regional facility‑based average. The heterogeneity itself is an important finding, reflecting substantial variation in obstetric populations, referral pathways, and clinical practices across the region.

Beyond methodological considerations, SSA experiences the world’s highest incidence of conditions that frequently necessitate cesarean delivery, including hypertensive disorders, obstructed labor, cephalopelvic disproportion, and fetal distress [69]. High fertility rates amplify the contribution of first-time deliveries [70], in contrast to lower-fertility settings where repeat cesareans predominate [71]. The continued high prevalence of adolescent pregnancy further compounds risk; incomplete pelvic maturation increases the likelihood of cephalopelvic disproportion and prolonged labor, thereby elevating PCS rates [6,72].

Robson classification analysis showed that low-risk groups were the dominant drivers of PCS. Group 1 (nulliparous, term, singleton, cephalic, spontaneous labor) alone accounted for 37.1% of all PCS, far exceeding the WHO reference contribution of 9.8%, while Group 3 contributed 34.1% compared with the WHO benchmark of 3%. Collectively, nulliparous pregnancies (Groups 1, 2, and 6) represented 52.9% of PCS. Observed rates within Groups 1 (32.6%), 3 (23.2%), and 4 (56.5%) exceeded WHO reference values (9.8%, 3.0%, and 23.7%, respectively), whereas Group 6 (63.8%) remained below the benchmark of 78.5% [67]. These patterns may suggest increased use of cesarean section among women classified within traditionally low-risk Robson groups; however, caution is warranted. The predominance of Group 1 is not unique to SSA. For example, a hospital-based study from China reported that Robson Group 1 accounted for 40.7% of all cesarean sections [73]. Interpretation of these findings is limited by the sparse and inconsistent reporting of maternal comorbidities and obstetric complications across the included studies. Important clinical factors such as hypertensive disorders, diabetes, HIV, placenta previa/accreta, fetal growth restriction, suspected macrosomia, chorioamnionitis, previous uterine surgery, and severe anemia were infrequently reported, preventing adjustment of PCS rates for underlying maternal and fetal risk differences [74]. Consequently, interpretations of potential cesarean section overuse in low-risk Robson groups should be made cautiously, as unmeasured maternal and obstetric risk factors may not have been fully captured by the classification system. Tertiary hospitals, which comprised the majority of included studies, also receive a disproportionate number of complicated pregnancies referred from lower-level facilities. Such referral patterns may contribute to elevated PCS rates even among women classified within low-risk Robson groups and may partially explain the substantial heterogeneity observed across studies [75].

Provider-initiated cesareans (Robson Groups 2 and 4) accounted for 36.6% of PCS, with 26.9% performed as prelabor cesareans and 9.7% following induction of labor. Prelabor cesareans were more frequent among nulliparous women (21.5%) than multiparous women (15.1%), consistent with greater clinical uncertainty, unfavorable cervical status, and perceived risk of prolonged labor in first-time mothers [76]. Ten studies reporting onset-of-labor subcategories showed markedly higher cesarean rates after spontaneous labor (18.8% in Groups 1 and 3 combined) than after induction (31.8%) or prelabor cesarean (89.8%). These findings can be explained by systemic gaps in intrapartum care. Delayed referrals from rural primary facilities, driven by transportation barriers, socioeconomic disparities, and weak infrastructure, often result in women arriving at tertiary centers with advanced complications, converting potentially manageable labors into emergency cesareans [77]. Additional contributors include limited provider training in active labor management, shortages of skilled staff and monitoring equipment, inconsistent use of partographs and evidence-based protocols, and defensive clinical practices aimed at minimizing perceived litigation or adverse outcomes [2,78].

The pooled PCS rate in SSA is comparable to estimates from Latin America (19.3%) [79], Peru (17.4%) [80], Iran (20.9%) [81], and Lithuania (21.6%) [82], yet lower than those reported in Türkiye (28.8%) [83], Brazil (28%) [84] and China (41.4%) [73]. Numerical similarity, however, masks profound contextual differences. In Latin America and other middle-income settings, elevated PCS rates are frequently driven by non-medical factors such as maternal request, provider preference, private-sector incentives, and over-medicalization [85]. In SSA, most cesareans are performed for emergency, life-saving indications amid restricted access to timely obstetric surgery and delayed presentation from rural areas [2].

Discrepancies with Türkiye, and Brazil may be partly explained by historical cesarean trends, while demographic context is relevant in China. In Türkiye and Brazil, cesarean section rates have remained substantially higher than vaginal birth rates for many years, and relatively low vaginal birth after cesarean (VBAC) rates (approximately 20%) may contribute to the persistence of repeat cesarean deliveries and concerns regarding potential overuse of cesarean section in some settings [86–89]. In China, annual deliveries declined by 24.5% from 2019 to 2023 while maternal age increased significantly [73], illustrating how fertility policy shifts alter the obstetric population profile in ways that differ fundamentally from SSA's high-fertility context. SSA reports substantially higher VBAC rates (60–80%), which, combined with higher fertility, may shift a greater proportion of the cesarean burden toward primary rather than repeat procedures [90].

Compared with lower PCS settings such as Egypt (11%) [91], Suriname (11%) [92], Malaysia (13.4%) [93], Canada (13.7%) [94], and Spain (15.7%) [95], the SSA estimate is notably higher. These countries may generally benefit from stronger emergency obstetric infrastructure, reliable access to blood products and surgical supplies, and more consistent adherence to standardized labor protocols. In SSA, systemic constraints, including shortages of skilled personnel, inconsistent partograph use, delayed referrals, and variable provider thresholds, likely contribute to higher intervention rates even in low-risk groups [2,77]. Maternal preferences, such as fear of labor pain, pelvic floor injury, or impacts on sexual function, may also play a modest role in some settings [96].

Among the subgroup analyses, cesarean section cost-exemption policy was the only study-level characteristic associated with a statistically significant difference in PCS prevalence. Countries with universal or near-universal cesarean section cost exemption had the highest pooled PCS prevalence (19.4%), followed by countries with partial exemption policies (17.7%), whereas countries without structured exemption policies had the lowest prevalence (14.9%). A possible explanation is that removing financial barriers improves access to medically indicated cesarean delivery and other emergency obstetric services. Previous studies have shown that out-of-pocket costs can delay care-seeking and limit access to essential maternal health services in low-resource settings [98], potentially contributing to lower utilization of cesarean section where cost-exemption policies are absent or limited [97]. However, this finding should be interpreted with caution because the number of studies in some subgroups was limited, particularly in countries without structured exemption policies, and considerable heterogeneity remained within subgroups. Furthermore, variations in health-system financing, availability of obstetric services, referral pathways, and access to comprehensive emergency obstetric care may have contributed to the observed differences in PCS prevalence across health facilities in the region [99].

Despite subgroup analyses, substantial heterogeneity remained (I² = 99.68%). The 95% prediction interval (8.4%–36.8%) further illustrates the wide range of possible true PCS rates across different SSA facilities, underscoring the limited generalizability of the pooled estimate alone. Heterogeneity in cesarean section studies is a recognized challenge globally. The WHO's endorsement of the Robson classification was intended to provide a standardized framework for meaningful comparison across settings, yet considerable between-facility variation persists even when Robson classification is applied. Beyond unmeasured maternal comorbidities and obstetric risk factors, this variability likely reflects the substantial diversity of health systems and service delivery contexts across SSA. Included studies were conducted across facilities with different referral roles, case mixes, resource availability, staffing patterns, and clinical practices. Regional and country-level differences in labor management protocols, induction practices, fetal monitoring availability, partograph utilization, VBAC policies, and diagnostic thresholds for labor dystocia or fetal compromise may further contribute to the observed variation [100]. The persistence of substantial heterogeneity despite subgroup analyses and univariable meta-regression indicates that important sources of between-study variability remain unexplained. This suggests that multiple interacting clinical, institutional, and health-system factors that were not captured by the available study-level covariates may influence PCS rates across the region. These contextual factors, combined with uneven distribution of comorbidities across regions and facility types, may explain both the elevated PCS rates in low-risk Robson groups and the persistent heterogeneity [101].

Overall, while cesarean section remains an essential life-saving intervention in SSA, the disproportionate contribution of low-risk Robson groups to PCS, interpreted in the context of sparse comorbidity data and heterogeneous practice patterns, underscores the need for targeted improvements. Routine use of the Robson classification for local audits, strengthened referral pathways, consistent adoption of evidence-based labor management protocols, improved documentation of maternal comorbidities and indications for cesarean delivery, and continued efforts to reduce financial barriers to emergency obstetric care may help optimize cesarean section use and improve maternal and neonatal outcomes across the region.

### Strengths and limitations

This review has several notable strengths. To our knowledge, it is the first systematic review and meta-analysis to comprehensively estimate the pooled prevalence of primary cesarean section (PCS) in Sub-Saharan Africa using the Robson Ten-Group Classification System. By specifically focusing on primary rather than overall cesarean delivery, the study provides more nuanced insight into the epidemiology of first-time cesarean birth and the obstetric groups contributing most substantially to the cesarean burden in the region. The use of the Robson classification enabled standardized assessment across studies and facilitated identification of high-contributing obstetric subgroups, thereby enhancing the clinical relevance of the findings. In addition, the inclusion of a large cumulative sample drawn from multiple countries across Sub-Saharan Africa strengthens the regional representativeness of the analysis and improves the precision of pooled estimates. The robustness of the findings was further supported through sensitivity analyses, subgroup analyses, meta-regression, prediction interval estimation, and formal assessment of publication bias.

Nevertheless, several limitations should be considered when interpreting the findings. First, substantial heterogeneity was observed across included studies, and although subgroup analyses and meta-regression were undertaken, the sources of between-study variability could not be fully explained. This heterogeneity likely reflects differences in study populations, institutional case mix, referral pathways, obstetric practices, and healthcare system capacity across settings. Second, all included studies were institution-based, which may limit the generalizability of findings to the broader population, particularly in rural and underserved communities where access to facility-based delivery remains limited. Furthermore, most included studies were conducted in secondary and tertiary referral hospitals, which often manage disproportionately high-risk pregnancies and may therefore overestimate the true burden of PCS compared with the general obstetric population. Third, most studies relied on retrospective review of medical records, introducing the potential for incomplete documentation, reporting inaccuracies, and variability in data quality. Fourth, important clinical variables, including maternal comorbidities, obstetric complications, indications for cesarean delivery, referral status, and severity of maternal condition at presentation, were inconsistently reported. This limited the ability to assess the appropriateness of cesarean use, adjust for differences in maternal risk profiles, or determine the extent to which elevated PCS rates reflected clinical need rather than practice variation. Fifth, the evidence base was geographically uneven, with studies originating from a limited number of countries and predominantly from higher-level facilities. Consequently, the findings may not fully capture variation in PCS practice across all Sub-Saharan African settings. Finally, the operational definition of PCS excluded women in Robson groups 7–10 because previous cesarean status could not be determined consistently across studies. This may have resulted in underestimation of the true prevalence of primary cesarean section.

## Conclusion

Routine implementation of the Robson classification for clinical audits, strict adherence to evidence‑based labor management protocols, and targeted interventions focusing on Groups 1 and 2 are critical for promoting the rational use of CS and improving maternal and neonatal outcomes in the region.

The rate of primary cesarean section (PCS) based on the Robson classification in Sub-Saharan Africa (SSA) exceeded the WHO reference value, with nearly half of the procedures originating from Robson groups 1 and 2, and more than one-third attributable to group 1 alone. This pattern suggests a potential overuse of cesarean delivery among low-risk women. This pattern raises concern for possible overuse among low‑risk women, though unmeasured comorbidities and referral bias cannot be excluded. PCS rates were notably higher in Groups 2B and 4B, and spontaneous labor among both nulliparous and multiparous women was associated with higher PCS rates compared to induction or prelabor cesarean, indicating possible deficiencies in intrapartum care and labor management. The considerable unexplained heterogeneity across studies, illustrated by a wide prediction interval (8.4%−36.8%), highlights the influence of contextual factors and the need for setting-specific investigations.

Routine implementation of the Robson classification for clinical audits, strict adherence to evidence-based labor management protocols, and targeted interventions focusing on Groups 1 and 2 are critical for promoting the rational use of CS and improving maternal and neonatal outcomes in the region.

### Recommendations

Based on the findings of this review, several actions are warranted to optimize PCS use in SSA.First, interventions should prioritize Robson groups 1 and 2. Strengthening adherence to evidence-based labor monitoring and management protocols for women in these groups is crucial to prevent unnecessary CSs. Second, the disproportionately high rates of prelabor cesarean deliveries in Groups 2B and 4B underscore the need for stricter justification and regular clinical audits of elective procedures. The consistent application of standardized induction protocols may help reduce unwarranted prelabor cesareans when induction is a safe alternative. Third, the higher frequency of cesarean deliveries following spontaneous labor compared to induction suggests potential deficiencies in intrapartum care. Enhancing providers’ skills in labor monitoring, timely decision-making, and appropriate interventions could help decrease unnecessary cesareans. Finally, the substantial heterogeneity observed across studies indicates that context-specific factors, such as provider attitudes, institutional practices, and women’s preferences, may influence cesarean use. Further primary research is needed to explore these contextual determinants and guide tailored interventions across different healthcare settings in the region.

## Supporting information

S1 File PRISMA Checklist 2020. (PDF)

S2 File Search strategy. (PDF)

S3 File Quality assessment of the included studies. (PDF)

S4 File Descriptive summary of the key characteristics of the studies included. (DOCX)
