# Supplementary material for: Primary cesarean section in Sub-Saharan Africa: A systematic review and meta-analysis using the Robson Ten-Group Classification System
Source: PLoS One. 2026 Jul 30;21(7):e0354911. doi: 10.1371/journal.pone.0354911 (PMC13422873; doi:10.1371/journal.pone.0354911)
Supplement: S1 File — (PDF) [file pone.0354911.s001.pdf]

PRISMA 2020 Checklist

Section and Topic	Item #	Checklist item	Location where item is reported
**TITLE**			
Title	1	Primary cesarean section rate in sub-Saharan Africa: a systematic review and meta-analysis using the Robson Ten-Group Classification System	
**ABSTRACT**			
Abstract	2	**Background**: Overuse of cesarean section contributes significantly to perinatal mortality in sub-Saharan Africa. Primary cesarean sections are frequently overused, with rates varying across countries. The Robson classification system is the global standard for monitoring cesarean section rates. Evidence on the pooled rate of PCS using the Robson Ten-Group Classification is limited in sub-Saharan Africa.<br>**Methods**: We conducted a systematic review and meta-analysis. We searched PubMed, Scopus, ScienceDirect, Google Scholar, Google, and ResearchGate for studies published between 2001 and February 22, 2025. Data were extracted into Microsoft Excel and analyzed in Stata 17. A random-effects model was applied to calculate the pooled rate. Study quality was assessed using the Joanna Briggs Institute Critical Appraisal Checklist for prevalence studies. Publication bias was assessed using funnel plots and Egger's test. Heterogeneity was assessed using the I^2 statistic.<br>**Results**: The pooled PCS rate in SSA was 19.1% (95% CI: 16.6-21.6). Robson groups 1 and 2 were the largest contributors (47.5%), with Group 1 alone contributing 37.1%. Primary cesarean section was more common after spontaneous labor than after induction or elective procedures, in both nulliparous and multiparous women. Significant heterogeneity was observed across studies ($I^2 = 99.64\%$, $p < 0.001$). No significant publication bias was identified.<br>**Conclusion**: The primary cesarean section rate is high in SSA, with Robson Groups 1 and 2 being the largest contributors. High rates of prelabor cesarean and elevated rates following spontaneous labor suggest potential overuse and gaps in labor management. Stricter adherence to evidence-based protocols and routine use of the Robson classification for clinical audits are essential to improving obstetric care.	
**INTRODUCTION**			
Rationale	3	Although PCS rates are a global maternal health priority, and standardized monitoring is recommended (22), evidence from SSA remains scarce. To our knowledge, this is the first systematic review and meta-analysis to determine PCS rates in SSA using the Robson classification.	
Objectives	4	What is the rate of primary cesarean section in sub-Saharan Africa according to the Robson classification?	
**METHODS**			
Eligibility criteria	5	Inclusion: observational studies using Robson classification in SSA, in English, any year.	

PRISMA 2020 Checklist

Section and Topic	Item #	Checklist item	Location where item is reported
		Exclusion: reviews, case reports, qualitative, incomplete Robson data, misclassification, sampling issues.	
Information sources	6	We searched Scopus, PubMed, ScienceDirect, Google Scholar, ResearchGate, and Google up to 22 February 2025.	
Search strategy	7	The search strategy was guided by the CoCoPop approach (Condition, Context, and Population) and utilized free-text keywords combined with Boolean operators such as "OR", "AND", and "NOT". We also assessed the relevance of each search term. The search strategy included terms related to "cesarean section" or "cesarean section", such as "C-section", "cesarean delivery", "cesarean delivery", "cesarean birth", "cesarean birth", "operative delivery", and "operative birth." Terms related to the Robson Classification, such as "Robson", "Modified Robson", "Robson Ten-Group", "Robson Ten Group", "Robson's Ten-Group", "Robson Criteria", "Robson Classification", "Robson Classification System", "Robson Classification Method", "Robson System", "Ten Group Classification System", "10-Group Classification System", "Robson TGCS", "RTGS", and "TGCS" were included. Terms indicating the study setting were also used such as "Africa", "Sub-Saharan Africa", and using the name of each Sub-Saharan African Country whenever the search conditions of the databases allowed.	
Selection process	8	Three reviewers (KYW, BAM, GMA) screened records independently. 3620 records were identified by the search strategy and were imported into EndNote 21. After removing duplicate and those in languages other than English with no translation available, 3234 records were screened by their title and abstract. 46 records were kept for full-text review against inclusion criteria. Eligible records were then checked in detail for complete outcome measurement and other issues regarding the outcome. Finally, 25 studies were included for this review. Discrepancies were resolved through discussion and consensus.	
Data collection process	9	Three authors (KYW, BAM, GMA) independently extracted data using an Excel abstraction form. When data were missing or unclear, we attempted to contact study authors by email. Disagreements in data extraction were resolved by discussion within the review team. We assessed interrater agreement using kappa statistics.	
Data items	10	For each study, extracted variables included: first author, publication year, year of data collection, country, income level, SSA subregion, study design, sampling technique, study setting, facility level (primary, secondary, or tertiary), facility type (public or private), study period, total sample size, PCS sample size, size of Robson Groups 1–10, number of women undergoing CS in each group, cost of cesarean services.	
Study risk of bias assessment	11	The quality of included studies was assessed using the Joanna Briggs Institute (JBI) critical appraisal checklist for observational studies. The checklist has nine items, yielding scores from 0–9. Studies were categorized as low quality (0–4), medium quality (5–7), or high quality (8–9). Only studies scoring 5 or above (medium or high quality) were included. Four authors (KYW, EBW, BAM, and MAL) independently evaluated study quality. Discrepancies were resolved by averaging reviewer scores.	
Effect measures	12	Pooled prevalence rates with 95% confidence intervals	
Synthesis methods	13a	The process for deciding which studies were eligible for synthesis began with importing all identified records into EndNote 21 to remove duplicates. Three reviewers independently screened the titles and abstracts, followed by a full-text review of the remaining articles against a predefined set of inclusion and exclusion criteria. The inclusion criteria required studies to be observational, use the Robson classification, be conducted in sub-Saharan Africa, be published or unpublished in English, and have no date restrictions. Studies were excluded for reasons such as a lack of full-text access, being a review or qualitative study, incomplete outcome measurements, missing total numbers for each Robson group, or data inconsistencies. Any discrepancies in the screening process were resolved through discussion and consensus among the reviewers.	
	13b	Data was extracted from the included studies into a Microsoft Excel abstraction form. Key variables extracted included the first author, publication year, country, study design, and sample sizes for the total population and each Robson group. To handle missing or unclear data, the authors attempted to contact the original study authors via email. The extracted data was then exported to Stata 17.0 for statistical analysis. Standard errors for the primary cesarean section (PCS) rates were calculated using a binomial distribution.	
	13c	The results of the meta-analysis were presented using a combination of methods. Pooled estimates with 95% confidence intervals were visually displayed using forest plots. Additional results were presented in tables and textual summaries. A PRISMA flow diagram was also used to document the study selection process.	

Section and Topic	Item #	Checklist item	Location where item is reported
	13d	he authors used a random-effects model (DerSimonian-Laird method) to calculate the pooled rate of primary cesarean sections. This model was chosen to account for the variability that exists between studies. To identify the presence and extent of statistical heterogeneity, they used the I^2 statistic, with values of 25%, 50%, and 75% indicating low, moderate, and high heterogeneity, respectively. They also assessed for publication bias using funnel plots and Egger's regression test. All statistical analyses were conducted using Stata 17.0.	
	13e	To explore possible causes of heterogeneity among study results, the authors of the manuscript used subgroup analyses. These analyses were based on study period, sampling technique, study design, institutional type and level, country income level, and region.	
	13f	The authors conducted a leave-one-out sensitivity analysis to assess the robustness of the synthesized results. This method involved systematically removing one study at a time to determine its influence on the overall pooled estimate of the primary cesarean section rate.	
Reporting bias assessment	14	The authors of the manuscript assessed for risk of bias due to missing results by using funnel plots and Egger's regression test to evaluate publication bias. A p-value of less than 0.05 from Egger's test was considered to indicate significant publication bias. The results of these tests did not find significant evidence of publication bias.	
Certainty assessment	15	The authors assessed the methodological quality of the included studies using the Joanna Briggs Institute (JBI) Critical Appraisal Checklist for prevalence studies. The checklist has nine items, and studies were scored from 0 to 9. Studies that scored 5 or above (medium or high quality) were included in the meta-analysis. Four authors independently evaluated the study quality, and any discrepancies were resolved by discussion or by averaging reviewer scores. All 25 included studies were found to have medium to high methodological quality, with scores ranging from seven to nine.	
**RESULTS**			
Study selection	16a	The search for studies identified 3,620 records. After removing 345 duplicates and 45 non-English studies, 3,234 records remained. Full-texts were not available for 22 records and another 3,168 studies were excluded by their title and abstract. The remaining 44 full-text articles were found eligible. Of these, 19 were excluded for reasons such as incomplete outcome measurements, not reporting the size of each Robson group, and data inconsistencies. finally, 25 studies met the inclusion criteria and were included in the meta-analysis. The PRISMA flow diagram was shown in Figure 1.	
	16b	19 records were excluded for the following reasons of<br>• One article for not reporting each Robson group completely (1)<br>• Thirteen studies for not reporting the total number of women/births in each Robson (2-14)<br>• Two studies for misclassifying the Robson groups/classification out of the standard (15, 16)<br>• Three studies for incorrect calculation across Robson groups (17-19)	
Study characteristics	17	The following 25 studies were included in the meta-analysis.<br>1. **(20):** A prospective, consecutive study of 4,004 women at a public tertiary hospital in Ethiopia, using Robson classification for cesarean delivery analysis.<br>2. **(21):** A retrospective convenience sampling study of 28,376 women at a public primary and secondary hospital in Mali, using Robson classification for cesarean delivery analysis.<br>3. **(22):** A retrospective, census-based study of 4,200 women at a public tertiary hospital in Ethiopia, using Robson classification for cesarean delivery analysis.<br>4. **(23):** A retrospective, census-based study of 20,270 women at a public tertiary hospital in Ghana, using Robson classification for cesarean delivery analysis.<br>5. **(24):** A retrospective, census-based study of 447 women at a private tertiary hospital in Nigeria, using Robson classification for cesarean delivery analysis.<br>6. **(25):** A retrospective, census-based study of 1,975 women at a public tertiary hospital in Sierra Leone, using Robson classification for	

Section and Topic	Item #	Checklist item	Location where item is reported
		cesarean delivery analysis.<br>7. **(26):** A retrospective, census-based study of 5,886 women at both public and private tertiary hospitals in Ethiopia, using Robson classification for cesarean delivery analysis.<br>8. **(27):** A prospective, systematic random study of 721 women at both public and private primary, secondary, and tertiary hospitals in Ethiopia, using Robson classification for cesarean delivery analysis.<br>9. **(28):** A retrospective, consecutive study of 3,030 women at a private tertiary hospital in Somalia, using Robson classification for cesarean delivery analysis.<br>10. **(29):** A retrospective, purposive study of 4,771 women at both public and private primary, secondary, and tertiary hospitals in Sierra Leone, using Robson classification for cesarean delivery analysis.<br>11. **(29) :** A retrospective, census-based study of 2,525 women at a public tertiary hospital in Kenya, using Robson classification for cesarean delivery analysis.<br>12. **(30) :** A retrospective, census-based study of 1,203 women at a private tertiary hospital in Ethiopia, using Robson classification for cesarean delivery analysis.<br>13. **(31) :** A prospective convenience sampling study of 35,287 women at a public primary, secondary, and tertiary hospital in Senegal, using Robson classification for cesarean delivery analysis.<br>14. **(32) :** A prospective convenience sampling study of 993 women at a public tertiary hospital in Ethiopia, using Robson classification for cesarean delivery analysis.<br>15. **(33) :** A retrospective convenience sampling study of 5,442 women at both public and private primary, secondary, and tertiary hospitals in Benin, using Robson classification for cesarean delivery analysis.<br>16. **(34) :** A retrospective, census-based study of 2,266 women at a public primary and secondary hospital in Guinea, using Robson classification for cesarean delivery analysis.<br>17. **(35) :** A retrospective, census-based study of 137,094 women at a public tertiary hospital in Tanzania, using Robson classification for cesarean delivery analysis.<br>18. **(36) :** A retrospective, census-based study of 56,314 women at a public tertiary hospital in Tanzania, using Robson classification for cesarean delivery analysis.<br>19. **(37) :** A retrospective, consecutive study of 556 women at a public tertiary hospital in Nigeria, using Robson classification for cesarean delivery analysis.<br>20. **(38) :** retrospective, purposive study of 1,276 women at a public tertiary hospital in Uganda, using Robson classification for cesarean delivery analysis.<br>21. **(39) :** A retrospective, census-based study of 3,079 women at a public primary and secondary hospital in Nigeria, using Robson classification for cesarean delivery analysis.<br>22. **(40) :** A prospective, census-based study of 2,139 women at a public tertiary hospital in Nigeria, using Robson classification for cesarean delivery analysis.<br>23. **(41) :** A retrospective, census-based study of 1,295 women at a public tertiary hospital in Nigeria, using Robson classification for cesarean delivery analysis.<br>24. **(42) :** A retrospective, purposive study of 3,183 women at a public tertiary hospital in Uganda, using Robson classification for cesarean delivery analysis.<br>25. **(43) :** A retrospective, census-based study of 3,012 women at a private tertiary hospital in Tanzania, using Robson classification for	

Section and Topic	Item #	Checklist item	Location where item is reported
		cesarean delivery analysis.	
Risk of bias in studies	18	Present assessments of risk of bias for each included study.<br>Based on the JBI quality assessment provided in the "Supplementary file 2", here are the risk of bias assessments for each included study. The total score for each study is out of a possible 9, with 'Y' indicating "Yes" and 'N' indicating "No".<br>The questions used for the assessment are:<br>1. Was the sample frame appropriate to address the target population?<br>2. Were study participants sampled in an appropriate way?<br>3. Was the sample size adequate?<br>4. Were the study subjects and the setting described in detail?<br>5. Was the data analysis conducted with sufficient coverage of the identified sample?<br>6. Were valid methods used for the identification of the condition?<br>7. Was the condition measured in a standard, reliable way for all participants?<br>8. Was there appropriate statistical analysis?<br>9. was the response rate adequate, and if not, was the low response rate managed appropriately?	
Results of individual studies	19	Magnitude of primary cesarean section with effect size and confidence intervals was shown in the forest plot below.	

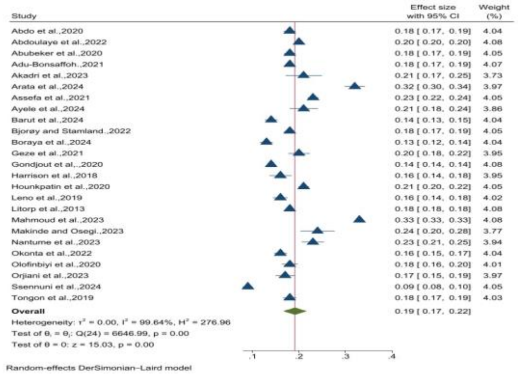

Section and Topic	Item #	Checklist item	Location where item is reported
Results of syntheses	20a	1. Abdo et al.,2020: Total score: 9. The study answered 'Yes' to all nine quality assessment questions.<br>2. Abdoulaye et al.,2022: Total score: 9. The study answered 'Yes' to all nine quality assessment questions.<br>3. Abubeker et al.,2020: Total score: 9. The study answered 'Yes' to all nine quality assessment questions.<br>4. Adu-Bonsaffoh.,2021: Total score: 9. The study answered 'Yes' to all nine quality assessment questions.<br>5. Akadri et al., 2023: Total score: 8. The study answered 'No' to question 3, "Was the sample size adequate?".<br>6. Arata et al., 2024: Total score: 9. The study answered 'Yes' to all nine quality assessment questions.<br>7. Assefa et al., 2021: Total score: 9. The study answered 'Yes' to all nine quality assessment questions.<br>8. Ayele et al., 2024: Total score: 8. The study answered 'No' to question 3, "Was the sample size adequate?".<br>9. Barut et al., 2024: Total score: 9. The study answered 'Yes' to all nine quality assessment questions.<br>10. Bjorøy and Stamland., 2022: Total score: 9. The study answered 'Yes' to all nine quality assessment questions.<br>11. Boraya et al., 2024: Total score: 9. The study answered 'Yes' to all nine quality assessment questions.<br>12. Geze et al., 2021: Total score: 9. The study answered 'Yes' to all nine quality assessment questions.<br>13. Gondjout et al., 2020: Total score: 9. The study answered 'Yes' to all nine quality assessment questions.<br>14. Harrison et al., 2018: Total score: 7. The study answered 'No' to question 2, "Were study participants sampled in an appropriate way?", and question 3, "Was the sample size adequate?".<br>15. Hounkpatin et al., 2020: Total score: 9. The study answered 'Yes' to all nine quality assessment questions.<br>16. Leno et al., 2019: Total score: 9. The study answered 'Yes' to all nine quality assessment questions<br>17. Litorp et al., 2013: Total score: 9. The study answered 'Yes' to all nine quality assessment questions.<br>18. Mahmoud et al., 2023: Total score: 9. The study answered 'Yes' to all nine quality assessment questions.<br>19. Makinde and Osegi., 2023: Total score: 8. The study answered 'No' to question 3, "Was the sample size adequate?".<br>20. Nantume et al., 2023: Total score: 9. The study answered 'Yes' to all nine quality assessment questions.<br>21. Okonta et al., 2022: Total score: 9. The study answered 'Yes' to all nine quality assessment questions.<br>22. Olofinbiyi et al., 2020: Total score: 9. The study answered 'Yes' to all nine quality assessment questions.<br>23. Orjiani et al., 2023: Total score: 9. The study answered 'Yes' to all nine quality assessment questions.<br>24. Ssennuni et al., 2024: Total score: 9. The study answered 'Yes' to all nine quality assessment questions.<br>25. Tongon et al., 2019: Total score: 9. The study answered 'Yes' to all nine quality assessment questions.	
	20b	The pooled rate of primary cesarean section (PCS) in sub-Saharan Africa was found to be 19.1%(95% CI: 16.6–21.6)<br>Significant statistical heterogeneity was observed across the studies indicated by an I^2 value of 99.64% and a p-value for Cochrane's Q of < 0.0001.	
	20c	Subgroup analyses that were performed did not explain the heterogeneity. The subgroups analyzed were based on: Study period, sampling technique, study design, institution type, level of institution, country income level, region.	

<table>
<tr><th>Section and Topic</th><th>Item #</th><th>Checklist item</th><th>Location where item is reported</th></tr>
<tr><td></td><td></td><td>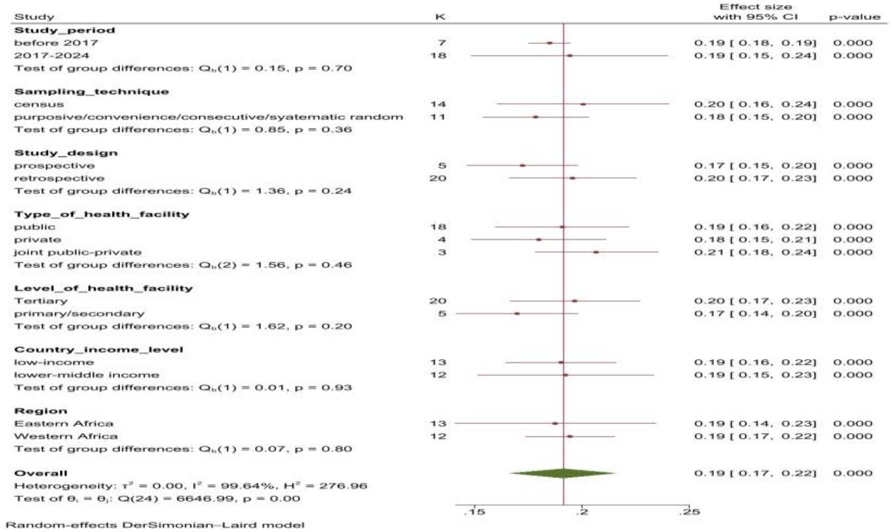
</td><td></td></tr>
<tr><td></td><td>20d</td><td>A leave-one-out sensitivity analysis was conducted to evaluate the impact of each study on the pooled estimate. The results showed that the pooled estimate was stable at 19.1% (95% CI: 16.6–21.6). The analysis demonstrated that excluding individual studies yielded effect sizes ranging from 18.4% (95% CI: 17.2–19.7) to 19.6% (95% CI: 17.1–22.1).
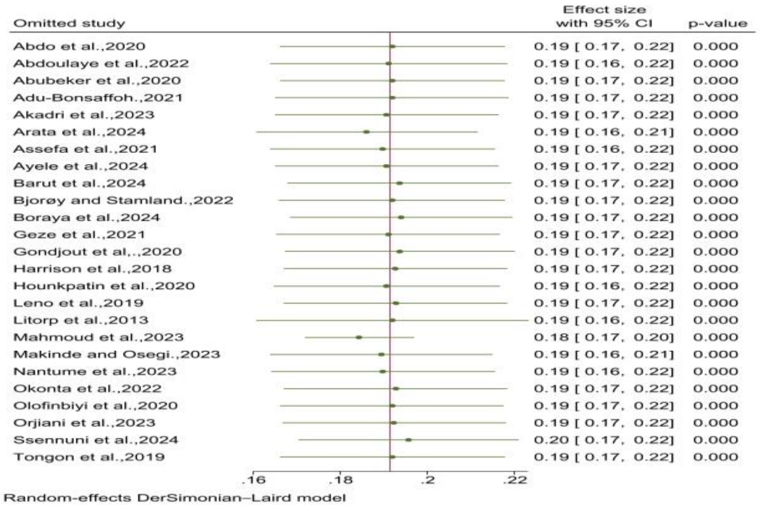
</td><td></td></tr>
<tr><td>Reporting biases</td><td>21</td><td>Publication bias was assessed via both a funnel plot and Egger’s test for small-study effects. Funnel plot assessment revealed no notable asymmetry. Egger’s test indicated no significant publication bias (β_1= 2.08, SE= 2.858, z= 0.73, p= 0.4657).</td><td></td></tr>
<tr><td>Certainty of</td><td>22</td><td>The certainty of evidence for the pooled primary cesarean section rate is low. This is primarily due to the observational nature of the included</td><td></td></tr>
</table>

PRISMA

PRISMA 2020 Checklist

Section and Topic	Item #	Checklist item	Location where item is reported
evidence		studies and the high, unexplained statistical heterogeneity (I^2=99.64%) found among the results, which indicate a lack of consistency.	
**DISCUSSION**			
Discussion	23a	The pooled primary cesarean section (PCS) rate of 19.1% found in this meta-analysis in Sub-Saharan Africa (SSA) is higher than the WHO's recommended rate of 11.7%, suggesting an overuse of the procedure. This finding is comparable to rates in other regions like Latin America (19.3%) but lower than countries with historically high rates such as Brazil (28%). The high rate in SSA may be influenced by the prevalence of tertiary and referral hospitals in the included studies, which handle more complex cases. The finding that Robson Groups 1 and 2 are the largest contributors to PCS (37.1% and 47.5% respectively) indicates that unnecessary cesareans may be occurring in low-risk women and points to possible gaps in labor management.	
	23b	The most significant limitation of the evidence is the high level of unexplained heterogeneity among the study results, which the subgroup analyses failed to account for. This suggests that there are other unmeasured contextual factors influencing the PCS rate. The review also did not assess the specific indications for cesarean sections, which limits the ability to draw conclusions on the reasons for the procedures.	
	23c	The authors acknowledge that their search strategy may not have captured all relevant studies due to variations in keywords and search terms. Additionally, the review did not account for other factors that could influence cesarean rates, such as provider-related factors, institutional policies, and patient preferences. The findings are based on available evidence, and a more comprehensive search might have yielded different results.	
	23d	The results have clear implications for practice, policy, and research. For practice, the high rates in low-risk groups (Groups 1 and 2) highlight the need for healthcare providers to adhere to evidence-based labor management protocols for nulliparous women to reduce unnecessary cesareans. In terms of policy, the data suggests that stricter justification and clinical auditing of elective prelabor cesareans are needed. Finally, for future research, the significant unexplained heterogeneity in the results indicates a need for studies to investigate specific contextual factors like provider decision-making and patient preferences that were not addressed in this review.	
**OTHER INFORMATION**			
Registration and protocol	24a	The review protocol was registered with the International Prospective Register of Systematic Reviews. The registration number is CRD42024623227	
	24b	The protocol was registered, and it can be accessed through the International Prospective Register of Systematic Reviews. The manuscript itself does not provide a direct link to the protocol.	
	24c	No amendments were done	
Support	25	No financial support received for this work	
Competing interests	26	Authors declared that they have no conflict of interest in this work	
Availability of data, code and other materials	27	Report which of the following are publicly available and where they can be found: template data collection forms; data extracted from included studies; data used for all analyses; analytic code; any other materials used in the review.	

References

1. Bello OO, Agboola AD. Utilizing the Robson 10-Group Classification System as an Audit Tool in Assessing the Soaring Caesarean Section Rates in Ibadan, Nigeria. J West Afr Coll Surg. 2022;12(1):64-9.
2. Ani VC, Ogabido CA, Ogelle MO, Ofojebe CJ, Okpala BC. An audit of caesarean section in a semi urban hospital in northern cross river state Nigeria utilizing the robson-10 criteria. World Journal of Advanced Research and Reviews. 2023;19(1):403-9.
3. Cyrille NNC, Alfred NI, Felix E, Louise T, Dupont KNJ. Cesarean Sections according to the Robson's Classification in Two University Hospitals of Yaoundé: Indications and Maternofetal Outcome. Open Journal of Obstetrics and Gynecology. 2023;13(11):1791-806.
4. Gebremichael MW, Meles K, Hagos H, Teka H, Gidey H, Yekoye A, et al. A Third of Cesarean Deliveries were Nulliparous Term Singleton Vertex in Tigray, Ethiopia: Employing the Robson Classification. East African Journal of Health Sciences. 2022;4(1):592-603.

5. Kanyi G, Khisa W, Ojwang S, Kilonzo M, Odawa F, Ondieki D, et al. Robson Classification for Caesarean Delivery Rates and Early Pregnancy Outcomes at Pumwani Maternity Hospital-Kenya. 2016.
6. Loue VA, Gbary EA, Koffi SV, Koffi AK, Traore M, Konan JK, et al. Analysis of caesarean rate and indications of university hospitals in sub-Saharan African developing countries using Robson classification system: the case of Cocody's hospital center, Abidjan-Cote d'Ivoire. Int J Reprod Contracept Obstet Gynecol. 2016;5(6):1773-7.
7. Makhanya V, Govender L, Moodley J. Utility of the Robson Ten Group Classification System to determine appropriateness of caesarean section at a rural regional hospital in KwaZulu-Natal, South Africa. S Afr Med J. 2015;105(4):292-5.
8. Mbaye M, Gueye M, Gueye MDN, Niang NKS, Moreau JC. Analysis of cesarean section rate according to Robson's classification in an urban health centre in Senegal. International Journal of Reproduction, Contraception, Obstetrics and Gynecology. 2015;4(4):1100-3.
9. Mulinganya G, Malembaka EB, Akonkwa ML, Mukendi DM, Birindwa EK, Balemba GM, et al. Applying the Robson classification to routine facility data to understand the Caesarean section practice in conflict settings of South Kivu, eastern DR Congo. PLOS ONE. 2020;15(9):e0237450-e.
10. Nana T, Nkwele F, Elong F, Tchounzou R, Essome H. Caesarean Section in a Referral Hospital in Sub-Saharan Africa: Frequency and Evaluation according to the Robson Classification. Arch Obstet Gynecol Reprod Med. 2023;6(2):243-50.
11. Ochejele S, Emoekpere H, Nkawu M, Alagh M. Robson classification of Caesarean sections at the Federal Medical Centre, Makurdi. Tropical Journal of Obstetrics and Gynaecology. 2021;38(3):261-5.
12. Ouattara A, Kiemtoré, S., Ouédraogo, I., Sawadogo, Y.A., Millogo, T., Bikienga, M., Kouanda, S. and Ouédraogo, C.M.R.-N. The Rate of Caesarean Sections in Burkina Faso's Regional and University Hospitals According to the Classification System of Robson's Ten Groups. Open Journal of Obstetrics and Gynecology. 2021(11):210-9.
13. Rukewe A, Orlam I, Akande A, Fatiregun AA. Distribution of cesarean delivery by Robson classification and predictors of postspinal anesthesia hypotension in Windhoek referral hospitals: A cross-sectional study. Niger J Clin Pract. 2022;25(2):178-84.
14. Tura AK, Pijpers O, de Man M, Cleveringa M, Koopmans I, Gure T, et al. Analysis of caesarean sections using Robson 10-group classification system in a university hospital in eastern Ethiopia: a cross-sectional study. BMJ Open. 2018;8(4):e020520.
15. Chukwu J, Egwu C, Chukwu C, Dotimi D. Analysis of Caesarean Delivery in General Hospitals in Sokoto State Using Robson's Ten Classification: A Cross-Sectional Study. Texila International Journal of Public Health. 2023;11:126-39.
16. Samba A MK. A Review of Caesarean Sections Using the Ten-group Classification System (Robson Classification) in the Korle-Bu Teaching Hospital (KBTH), Accra, Ghana. Gynecol Obstet (Sunnyvale) 6: 385. 2016.
17. Aduloju OP. Audit of Caesarean Section using the Modified Robson's Criteria at a Cottage Hospital in Port-Harcourt, Nigeria. Nigerian Stethoscope. 2024;6(2):38-43.
18. Chrifi Hassan AB, Boudallaa Ikram, Bititi Abderrahmane Amine and Soulaymani Abdelmajid. CAESAREAN SECTION RATES AT THE HOSPITAL MATERNITY: A CASE STUDY IN MOROCCO USING THE ROBSON CLASSIFICATION SYSTEM. Int J of Adv Res. 2022:497-506.
19. Kaboré C, Bocoum F, Kanyala/Kaboré E, Bonané/Thiéba B. Reporting Caesarean Delivery in district hospitals in Ouagadougou Using the Robson Classification System. Science et technique, série Sciences de la santé. 2018;4(2):45-52.
20. Abdo AA, Hinderaker SG, Tekle AG, Lindtjørn B. Caesarean section rates analysed using Robson's 10-Group Classification System: a cross-sectional study at a tertiary hospital in Ethiopia. BMJ open. 2020;10(10):e039098.
21. Abdoulaye SISSOKO IT, Seydou FANE, Amadou BOCOUM, Mohamed Yaya, DJIRE MAB, Amose KODIO, Siaka DIARRA, Amadou FOMBA, Tiounkani, THERA YTaNM. Interest in the Classification of Caesarean Section According to Robson at Teaching Hospital GABRIEL TOURE at Bamako, Mali. Gynecology & Reproductive Health. 2022;6(3):4.
22. Abubeker FA, Gashawbeza B, Gebre TM, Wondafrash M, Teklu AM, Degu D, et al. Analysis of cesarean section rates using Robson ten group classification system in a tertiary teaching hospital, Addis Ababa, Ethiopia: a cross-sectional study. BMC Pregnancy Childbirth. 2020;20(1):767.
23. Adu-Bonsaffoh K, Seffah J. Analysis Of Caesarean Sections Based On Robson Classification At A Tertiary Hospital In Ghana: A Cross-Sectional Pre-And Post-Intervention Study. Postgraduate Medical Journal of Ghana. 2021;10(1):47-53.
24. Akadri AA, Imaralu JO, Salami OF, Nwankpa CC, Adepoju AA. Robson classification of caesarean births: implications for reducing caesarean section rate in a private tertiary hospital in Nigeria. BMC Pregnancy Childbirth. 2023;23(1):243.
25. Arata M, Boyle S, Sgorbissa B, Tognon F, John-Cole V, Orsi M, et al. Caesarean sections, indications and outcomes: a cross-sectional study using the Robson classification in a tertiary hospital in Sierra Leone. BMJ Open. 2024;14(9):e081143.
26. Assefa E, Adem J, Gebrehiwot Y. Comparative analysis of cesarean section using the Robson's Ten-Group Classification System (RTCGS) in private and public hospitals, Addis Ababa, Ethiopia. Clinical Journal of Obstetrics and Gynecology. 2021;4:81-91.
27. Ayele M, Berta M, Zewdie A, Lake ES, Belayneh M. Analysis of caesarean delivery rates using Robson ten group classification system in North West Amhara referral hospitals, Ethiopia, 2022: A cross-sectional study. Scientific African. 2024;24:e02237-e.
28. Barut A, Erkok U, Hassan HB. Cross-sectional analysis of caesarean sections according to the Robson 10-group classification system in Somalia. BMJ Open. 2025;15(1):e086525.
29. Bjorøy S-A, Stamland G. Obstetrical Mapping and classification of Caesarean Sections according to the Robson Groups in eight hospital facilities in Sierra Leone: NTNU; 2022.
30. Geze S, Tura AK, Fage SG, van den Akker T. Can the Robson 10 Group Classification System help identify which groups of women are driving the high caesarean section rate in major private hospitals in eastern Ethiopia? A cross-sectional study. BMJ Open. 2021;11(8):e047206.

31. Gondjout TS, Gassama O, Diadhiou MT, Dieme MEF, Gueye M, Diouf AA, et al. Analysis of Cesarean Section Indications According to the Robson Classification in Surgical Maternities in Dakar, Senegal: About 9185 Cases. Journal of Gynecology and Obstetrics. 2020;8(5):135-40.
32. Harrison MS, Liyew T, Kirub E, Teshome B, Jimenez-Zambrano A, Muldrow M, et al. Use of cesarean birth at Mizan Tepi university teaching hospital, Mizan aman, Ethiopia. Midwifery. 2021;92:102860.
33. Hounkpatin B, Aboubakar M, Dangbemey P, Tognifode V, Schantz C, Dumont A, et al. Practice of the Caesarean Section in Four Maternities in Benin Using Robson Classification. Open Journal of Obstetrics and Gynecology. 2019;10(1):65-75.
34. Leno DW, Bah ME, Moumbagna JC, Millimouno TM, Lamah D, Delamou A, et al. Evaluation of caesarean section practices according to Robson's 10-group classification at a level two maternity ward in Conakry, Guinea. International Journal of Reproduction, Contraception, Obstetrics and Gynecology. 2019;8(11):4469.
35. Litorp H, Kidanto HL, Nystrom L, Darj E, Essén B. Increasing caesarean section rates among low-risk groups: a panel study classifying deliveries according to Robson at a university hospital in Tanzania. BMC Pregnancy Childbirth. 2013;13:107.
36. Mahmoud SA, Ussi AM, Khamis RS, Said SA, Okafor C, Mansab MR, et al. Stepping Towards Improved Audit Quality: The Outcome of Caesarean Section Using the Robson Ten Group Classification System at Mnazi Mmoja Hospital, Zanzibar. Journal of Gynecology and Obstetrics 2023, Volume 11, Page 73. 2023;11(3):73-9.
37. Makinde O, Osegi N. Towards Optimizing Caesarean Section: Robson Ten Group Analysis of Caesarean Section and It's Determinants in a Tertiary Hospital in South-South, Nigeria. Journal of Maternal and Child Health. 2023;8:682-95.
38. Nantume S, Baluku EM, Kwesiga D, Waiswa P. Factors Associated with High Rates of Caesarean Deliveries: A Cross Sectional Study Classifying Deliveries According to Robson in Mengo Hospital Kampala. Risk Manag Healthc Policy. 2023;16:2339-56.
39. Okonta PI, Fajola A, Umejiego C. An Analysis of Caesarean Sections in a Community Cottage Hospital in Nigeria's Niger Delta Using The Robson Classification. Niger Med J. 2022;63(2):91-7.
40. Olofinbiyi BA, Awoleke JO, Olaogun OD, Sunday A. Caesarean section audit: The use of the Robson's criteria in a teaching hospital with limited advanced fetal surveillance. International Journal of Innovative Research in Medical Science (IJIRMS). 2020;5(07).
41. Orjiani JC, Ocheke AN, Michael KC. Analysis of caesarean sections using robson ten group classification system at the Jos University Teaching Hospital. Highland Medical Research Journal. 2024;24(1):45-50.
42. Ssennuni E, Bongomin F, Akuma E, Lukujja K, Kule H, Opiro K, et al. Caesarean section rates in a tertiary teaching hospital in northern Uganda: a retrospective analysis using the robson ten group classification system. BMC Pregnancy Childbirth. 2024;24(1):489.
43. Tognon F, Borghero A, Putoto G, Maziku D, Torelli GF, Azzimonti G, et al. Analysis of caesarean section and neonatal outcome using the Robson classification in a rural district hospital in Tanzania: an observational retrospective study. BMJ Open. 2019;9(12):e033348.
