# Supplementary material for: Primary cesarean section in Sub-Saharan Africa: A systematic review and meta-analysis using the Robson Ten-Group Classification System
Source: PLoS One. 2026 Jul 30;21(7):e0354911. doi: 10.1371/journal.pone.0354911 (PMC13422873; doi:10.1371/journal.pone.0354911)
Supplement: S2 File — (PDF) [file pone.0354911.s002.pdf]

Database	Search Term
PubMed	(Robson[tw] OR “Modified Robson” [tw] OR “Robson Ten Group”[tw] OR “Robson Ten-Group” [tw] OR “Robson’s Ten-Group”[tw] OR “Robson Criteria”[tw] OR “Robson classification”[tw] OR “Robson classification system”[tw] OR “Robson classification method”[tw] OR “Robson System”[tw] OR “Ten Group Classification System”[tw] OR “Robson TGCS”[tw] OR RTGS[tw] OR TGCS[tw] OR RTGCS[tw]))<br>AND<br>(Ethiopia OR Uganda OR “Sierra Leone” OR Rwanda OR “South Africa” OR Nigeria OR Tanzania OR Congo OR “Democratic Republic of the Congo” OR Cameroon OR Ghana OR Angola OR Mali OR Malawi OR Gambia OR Benin OR Botswana OR “Burkina Faso” OR Burundi OR "Cabo Verde" OR “Central African Republic” OR Chad OR Comoros OR “Republic of Cote d'Ivoire” OR “Ivory Coast” OR “Equatorial Guinea” OR Eritrea OR Eswatini OR Swaziland OR Gabon OR Guinea OR “Guinea-Bissau” OR Kenya OR Lesotho OR Liberia OR Madagascar OR Mauritania OR Mauritius OR Mozambique OR Namibia OR Niger OR “Sao Tome and Principe” OR “Sao Tome” OR Senegal OR Seychelles OR Somalia OR “South Sudan” OR Sudan OR Togo OR Zambia OR Zimbabwe)
Scopus	(TITLE-ABS-KEY(“Cesarean Section”) OR TITLE-ABS-KEY(“Caesarean Section”) OR TITLE-ABS-KEY(C-Section) OR TITLE-ABS-KEY(“Cesarean Deliver*”) OR TITLE-ABS-KEY(“Caesarean Deliver*”) OR TITLE-ABS-KEY(“Caesarean Birth*”) OR TITLE-ABS-KEY(“Cesarean Birth”) OR TITLE-ABS-KEY(“Operative Delivery”) OR TITLE-ABS-KEY(“Operative Birth”))<br>AND<br>(TITLE-ABS-KEY(Robson) OR TITLE-ABS-KEY(“Modified Robson”) OR TITLE-ABS-KEY(“Robson Ten-Group”) OR TITLE-ABS-KEY(“Robson Ten Group”) OR TITLE-ABS-KEY(“Robson’s Ten-Group”) OR TITLE-ABS-KEY(“Robson Criteria”) OR TITLE-ABS-KEY(“Robson Classification”) OR TITLE-ABS-KEY(“Robson Classification System”) OR

	TITLE-ABS-KEY("Robson Classification Method") OR TITLE-ABS-KEY("Robson System") OR TITLE-ABS-KEY("Robson TGCS") OR TITLE-ABS-KEY(RTGS) OR TITLE-ABS-KEY(TGCS) OR TITLE-ABS-KEY(RTGCS))<br>AND<br>(TITLE-ABS-KEY(Ethiopia) OR TITLE-ABS-KEY(Uganda) OR TITLE-ABS-KEY("Sierra Leone") OR TITLE-ABS-KEY(Rwanda) OR TITLE-ABS-KEY("South Africa") OR TITLE-ABS-KEY(Nigeria) OR TITLE-ABS-KEY(Tanzania) OR TITLE-ABS-KEY(Congo) OR TITLE-ABS-KEY("Democratic Republic of Congo") OR TITLE-ABS-KEY(Cameroon) OR TITLE-ABS-KEY(Ghana) OR TITLE-ABS-KEY(Angola) OR TITLE-ABS-KEY(Mali) OR TITLE-ABS-KEY(Malawi) OR TITLE-ABS-KEY(Gambia) OR TITLE-ABS-KEY(Benin) OR TITLE-ABS-KEY(Botswana) OR TITLE-ABS-KEY("Burkina Faso") OR TITLE-ABS-KEY(Burundi) OR TITLE-ABS-KEY("Cabo Verde") OR TITLE-ABS-KEY("Central African Republic") OR TITLE-ABS-KEY(Chad) OR TITLE-ABS-KEY(Comoros) OR TITLE-ABS-KEY("Republic of Cote d'Ivoire") OR TITLE-ABS-KEY("Ivory Coast") OR TITLE-ABS-KEY("Equatorial Guinea") OR TITLE-ABS-KEY(Eritrea) OR TITLE-ABS-KEY(Eswatini) OR TITLE-ABS-KEY(Swaziland) OR TITLE-ABS-KEY(Gabon) OR TITLE-ABS-KEY(Guinea) OR TITLE-ABS-KEY("Guinea-Bissau") OR TITLE-ABS-KEY(Kenya) OR TITLE-ABS-KEY(Lesotho) OR TITLE-ABS-KEY(Liberia) OR TITLE-ABS-KEY(Madagascar) OR TITLE-ABS-KEY(Mauritania) OR TITLE-ABS-KEY(Mauritius) OR TITLE-ABS-KEY(Mozambique) OR TITLE-ABS-KEY(Namibia) OR TITLE-ABS-KEY(Niger) OR TITLE-ABS-KEY("Sao Tome and Principe") OR TITLE-ABS-KEY("Sao Tome") OR TITLE-ABS-KEY(Senegal) OR TITLE-ABS-KEY(Seychelles) OR TITLE-ABS-KEY(Somalia) OR TITLE-ABS-KEY("South Sudan") OR TITLE-ABS-KEY(Sudan) OR TITLE-ABS-KEY(Togo) OR TITLE-ABS-KEY(Zambia) OR TITLE-ABS-KEY(Zimbabwe))

**Science Direct**	((Cesarean OR Caesarean) AND (“Robson Classification” OR “Ten-Group” OR “Ten Group Classification” OR “10-Group Classification” OR TGCS) AND (Africa OR “Sub-Saharan Africa”))
Google Scholar	(“Cesarean section” OR “Caesarean section” OR “C-Section” OR “Cesarean deliver*” OR “Caesarean deliver*” OR “Caesarean Birth*” OR “Cesarean Birth*” OR “Operative Delivery” OR “Operative Birth”) AND (Robson OR “Modified Robson” OR “Robson Ten-Group” OR “Robson Ten Group” OR “Robson’s ten-group” OR “Robson Criteria” OR ”Robson classification” OR “Robson classification system” OR “Robson classification Method” OR “Robson System” OR “Ten Group Classification System” OR “Robson TGCS” OR RTGS OR TGCS OR RTGCS) AND (Ethiopia OR Uganda OR "Sierra Leone" OR Rwanda OR "South Africa" OR Nigeria OR Tanzania OR Congo OR "Democratic Republic of Congo" OR Cameroon OR Ghana OR Angola OR Mali OR Malawi OR Gambia OR Benin OR Botswana OR "Burkina Faso" OR Burundi OR "Cabo Verde" OR "Central African Republic" OR Chad OR Comoros OR " Republic of Cote d'Ivoire" OR "Ivory Coast" OR "Equatorial Guinea" OR Eritrea OR Eswatini OR Swaziland OR Gabon OR Guinea OR "Guinea-Bissau" OR Kenya OR Lesotho OR Liberia OR Madagascar OR Mauritania OR Mauritius OR Mozambique OR Namibia OR Niger OR "Sao Tome and Principe" OR "Sao Tome" OR Senegal OR Seychelles OR Somalia OR "South Sudan" OR Sudan OR Togo OR Zambia OR Zimbabwe)
Research Gate	(“Cesarean section” OR “Caesarean section”) AND (Robson OR “Ten-Group classification” OR “ 10-Group classification) AND Africa
Google	“Cesarean section” OR “Caesarean section”) AND “Robson classification” AND Africa
