# Supplementary material for: Primary cesarean section in Sub-Saharan Africa: A systematic review and meta-analysis using the Robson Ten-Group Classification System
Source: PLoS One. 2026 Jul 30;21(7):e0354911. doi: 10.1371/journal.pone.0354911 (PMC13422873; doi:10.1371/journal.pone.0354911)
Supplement: S3 File — (PDF) [file pone.0354911.s003.pdf]

	Study_ID	Q1	Q 2	Q3	Q4	Q5	Q6	Q7	Q8	Q9	Total score (9)
1.	Abdo et al.,2020	Y	Y	Y	Y	Y	Y	Y	Y	Y	9
2.	Abdoulaye et al.,2022	Y	Y	Y	Y	Y	Y	Y	Y	Y	9
3.	Abubeker et al.,2020	Y	Y	Y	Y	Y	Y	Y	Y	Y	9
4.	Adu-Bonsaffoh.,2021	Y	Y	Y	Y	Y	Y	Y	Y	Y	9
5.	Akadri et al.,2023	Y	Y	N	Y	Y	Y	Y	Y	Y	8
6.	Arata et al.,2024	Y	Y	Y	Y	Y	Y	Y	Y	Y	9
7.	Assefa et al.,2021	Y	Y	Y	Y	Y	Y	Y	Y	Y	9
8.	Ayele et al.,2024	Y	Y	N	Y	Y	Y	Y	Y	Y	8
9.	Barut et al.,2024	Y	Y	Y	Y	Y	Y	Y	Y	Y	9
10.	Bjorøy and Stamland.,2022	Y	Y	Y	Y	Y	Y	Y	Y	Y	9
11.	Boraya et al.,2024	Y	Y	Y	Y	Y	Y	Y	Y	Y	9
12.	Geze et al.,2021	Y	Y	Y	Y	Y	Y	Y	Y	Y	9
13.	Gondjout et al,.,2020	Y	Y	Y	Y	Y	Y	Y	Y	Y	9
14.	Harrison et al.,2018	Y	N	N	Y	Y	Y	Y	Y	Y	7
15.	Hounkpatin et al.,2020	Y	Y	Y	Y	Y	Y	Y	Y	Y	9
16.	Leno al.,2019	Y	Y	Y	Y	Y	Y	Y	Y	Y	9
17.	Litorp et al.,2013	Y	Y	Y	Y	Y	Y	Y	Y	Y	9
18.	Mahmoud et al.,2023	Y	Y	Y	Y	Y	Y	Y	Y	Y	9
19.	Makinde and Osegi.,2023	Y	Y	N	Y	Y	Y	Y	Y	Y	8
20.	Nantume et al.,2023	Y	Y	Y	Y	Y	Y	Y	Y	Y	9
21.	Okonta et al.,2022	Y	Y	Y	Y	Y	Y	Y	Y	Y	9
22.	Olofinbiyi et al.,2020	Y	Y	Y	Y	Y	Y	Y	Y	Y	9
23.	Orjiani et al.,2023	Y	Y	Y	Y	Y	Y	Y	Y	Y	9
24.	Ssennuni et al.,2024	Y	Y	Y	Y	Y	Y	Y	Y	Y	9
25.	Tongon et al.,2019	Y	Y	Y	Y	Y	Y	Y	Y	Y	9

Key: Y= yes; **N** = no

Question codes:

1. Was the sample frame appropriate to address the target population?

2. Were study participants sampled in an appropriate way?

3. Was the sample size adequate?

4. Were the study subjects and the setting described in detail?

5. Was the data analysis conducted with sufficient coverage of the identified sample?

6. Were valid methods used for the identification of the condition?

7. Was the condition measured in a standard, reliable way for all participants?

8. Was there appropriate statistical analysis?

9. was the response rate adequate, and if not, was the low response rate managed appropriately?
