# Supplementary material for: Primary cesarean section in Sub-Saharan Africa: A systematic review and meta-analysis using the Robson Ten-Group Classification System
Source: PLoS One. 2026 Jul 30;21(7):e0354911. doi: 10.1371/journal.pone.0354911 (PMC13422873; doi:10.1371/journal.pone.0354911)
Supplement: S4 File — (DOCX) [file pone.0354911.s004.docx]

| Study ID | Study period | Country | CSD service **^A^** | Country income level **^B^** | Study region **^C^** | Sampling technique | Type of institution **^D^** | Level of institution **^E^** | Study design **^F^** | Total women in labour | Women with no previous CS (n) | Cesarean delivered women | Robson Group 1 | | Robson Group 2 | | Robson Group 3 | | Robson Group 4 | | Robson Group 6 | |
| --- | --- | --- | --- | --- | --- | --- | --- | --- | --- | --- | --- | --- | --- | --- | --- | --- | --- | --- | --- | --- | --- | --- |
|  |  |  |  |  |  |  |  |  |  |  |  |  | Group size | CSD | Group size | CSD | Group size | CSD | Group size | CSD | Group size | CSD |
| Abdo et al.,2020 | 2019 | Ethiopia | 1 | 1 | 1 | consecutive | 1 | 1 | 1 | 4004 | 2881 | 731 | 1094 | 301 | 227 | 97 | 1356 | 227 | 158 | 68 | 46 | 38 |
| Abdoulaye et al.,2022 | 2013 | Mali | 1 | 1 | 2 | convenience | 1 | 1 | 2 | 28376 | 18075 | 5584 | 4391 | 2089 | 469 | 428 | 12138 | 2183 | 942 | 837 | 135 | 47 |
| Abubeker et al.,2020 | 2018 | Ethiopia | 1 | 1 | 1 | census | 1 | 1 | 2 | 4200 | 2844 | 768 | 1121 | 156 | 368 | 267 | 934 | 66 | 327 | 231 | 94 | 48 |
| Adu-Bonsaffoh.,2021 | 2013 | Ghana | 1 | 2 | 2 | census | 1 | 1 | 2 | 20270 | 12765 | 3624 | 3787 | 835 | 1502 | 1066 | 5455 | 561 | 1570 | 852 | 451 | 310 |
| Akadri et al.,2023 | 2022 | Nigeria | 1 | 2 | 2 | census | 2 | 1 | 2 | 447 | 283 | 96 | 86 | 21 | 38 | 32 | 136 | 23 | 18 | 15 | 5 | 5 |
| Arata et al.,2024 | 2021 | Sierra Leone | 1 | 1 | 2 | census | 1 | 1 | 2 | 1975 | 1519 | 641 | 559 | 238 | 71 | 58 | 763 | 249 | 80 | 63 | 46 | 33 |
| Assefa et al.,2021 | 2017 | Ethiopia | 1 | 1 | 1 | census | 3 | 1 | 2 | 5886 | 4427 | 1344 | 1773 | 532 | 425 | 304 | 1782 | 260 | 326 | 154 | 121 | 94 |
| Ayele et al.,2024 | 2022 | Ethiopia | 1 | 1 | 1 | Systematic Random | 3 | 3 | 1 | 721 | 468 | 154 | 198 | 66 | 48 | 24 | 157 | 32 | 51 | 23 | 14 | 9 |
| Barut et al.,2024 | 2023 | Somalia | 2 | 1 | 1 | Consecutive | 2 | 1 | 2 | 3030 | 1926 | 436 | 477 | 117 | 114 | 97 | 1188 | 103 | 122 | 101 | 25 | 18 |
| Bjorøy and Stamland.,2022 | 2021 | Sierra Leone | 1 | 1 | 2 | purposive | 3 | 3 | 2 | 4771 | 3709 | 844 | 1406 | 347 | 44 | 29 | 2108 | 379 | 89 | 49 | 62 | 40 |
| Boraya et al.,2024 | 2024 | Kenya | 1 | 2 | 1 | census | 1 | 1 | 2 | 2525 | 1561 | 330 | 444 | 8 | 284 | 171 | 604 | 3 | 186 | 121 | 43 | 27 |
| Geze et al.,2021 | 2020 | Ethiopia | 1 | 1 | 1 | census | 2 | 1 | 2 | 1203 | 987 | 244 | 197 | 71 | 27 | 19 | 690 | 114 | 72 | 39 | 1 | 1 |
| Gondjout et al,.,2020 | 2019 | Senegal | 1 | 2 | 2 | convenience | 1 | 3 | 1 | 35287 | 17679 | 4868 | 5893 | 1938 | 4658 | 863 | 3070 | 1020 | 2611 | 579 | 1447 | 468 |
| Harrison et al.,2018 | 2019 | Ethiopia | 1 | 1 | 1 | convenience | 1 | 1 | 1 | 993 | 787 | 159 | 300 | 58 | 56 | 21 | 365 | 59 | 57 | 14 | 10 | 7 |
| Hounkpatin et al.,2020 | 2016 | Benin | 1 | 2 | 2 | convenience | 3 | 3 | 2 | 5442 | 3354 | 1141 | 1118 | 416 | 238 | 171 | 1652 | 384 | 251 | 107 | 95 | 63 |
| Leno et al.,2019 | 2016 | Guinea | 1 | 1 | 2 | census | 1 | 2 | 2 | 2266 | 1466 | 365 | 467 | 109 | 121 | 69 | 712 | 98 | 101 | 53 | 65 | 36 |
| Litorp et al.,2013 | 2011 | Tanzania | 1 | 2 | 1 | census | 1 | 1 | 2 | 137094 | 97791 | 24200 | 42412 | 11585 | 773 | 512 | 52755 | 11059 | 814 | 591 | 1037 | 453 |
| Mahmoud et al.,2023 | 2022 | Tanzania | 1 | 2 | 1 | census | 1 | 1 | 2 | 56314 | 29462 | 18654 | 6170 | 4790 | 7975 | 2417 | 6524 | 5142 | 6997 | 4509 | 1796 | 1796 |
| Makinde and Osegi.,2023 | 2021 | Nigeria | 1 | 2 | 2 | consecutive | 1 | 1 | 2 | 556 | 380 | 136 | 85 | 30 | 13 | 10 | 252 | 74 | 24 | 19 | 6 | 3 |
| Nantume et al.,2023 | 2019 | Uganda | 1 | 1 | 1 | purposive | 1 | 1 | 2 | 1276 | 908 | 297 | 255 | 110 | 76 | 48 | 472 | 82 | 101 | 53 | 4 | 4 |
| Okonta et al.,2022 | 2018 | Nigeria | 1 | 2 | 2 | census | 1 | 2 | 2 | 3079 | 2484 | 483 | 877 | 231 | 249 | 102 | 1168 | 80 | 153 | 33 | 37 | 37 |
| Olofinbiyi et al.,2020 | 2015 | Nigeria | 1 | 2 | 2 | census | 1 | 1 | 1 | 2139 | 1493 | 395 | 569 | 176 | 105 | 42 | 700 | 121 | 68 | 15 | 51 | 41 |
| Orjiani et al.,2023 | 2018 | Nigeria | 1 | 2 | 2 | census | 1 | 1 | 2 | 1295 | 926 | 217 | 253 | 76 | 59 | 48 | 535 | 34 | 69 | 50 | 10 | 9 |
| Ssennuni et al.,2024 | 2020 | Uganda | 1 | 1 | 1 | purposive | 1 | 1 | 2 | 3183 | 2715 | 299 | 923 | 88 | 16 | 6 | 1719 | 185 | 18 | 10 | 39 | 10 |
| Tongon et al.,2019 | 2015 | Tanzania | 1 | 2 | 1 | census | 2 | 1 | 2 | 3012 | 2226 | 529 | 1128 | 309 | 58 | 35 | 974 | 147 | 45 | 25 | 21 | 13 |

“A” 1=free 2=paid

“B” 1=low-income 2=lower-middle income

“C” 1= Eastern Africa 2=Western Africa

“D” 1=public 2=private 3=both

“E” 1=tertiary 2=primary and secondary 3=primary, secondary and tertiary

“F” 1=prospective 2=retrospective
