# Supplementary material for: Primary cesarean section in Sub-Saharan Africa: A systematic review and meta-analysis using the Robson Ten-Group Classification System
Source: PLoS One. 2026 Jul 30;21(7):e0354911. doi: 10.1371/journal.pone.0354911 (PMC13422873; doi:10.1371/journal.pone.0354911)
Supplement: Attachment — Submitted filename: Response to Reviewers.docx [file pone.0354911.s007.docx]

Response to Reviewers

PONE-D-25-55783

Primary cesarean section rate in sub-Saharan Africa: a systematic review and meta-analysis using the Robson Ten-Group Classification System

**Dear Reviewer #1 and Editor,**

We sincerely thank Reviewer #1 for the thorough, constructive, and highly detailed comments, which have substantially improved the transparency, reproducibility, and interpretive rigor of the manuscript. Below we address each point exactly as raised. Where the reviewer combined several related issues into one comment, we have broken them down into specific sub-points for precision.

**Major comments**

**Introduction**

1. **Reviewer comment**: “The Introduction is generally clear but could be more concise, ie reduce narrative text; keep 2-3 short paragraphs ending with a precise objective and (ideally) a brief statement of the analytical approach”

**Author response**: The introduction has been shortened and restructured. The final paragraph now clearly states the study objective and the analytical approach using the Robson Ten-Group Classification System for pooled estimation of primary cesarean section rates. (See page 3, lines 42-62).

**Methods**

2. **Reviewer comment:**

“While databases, timeframe, software, and random-effects model are described, offer a greater detail to allow replication. e.g. clarify: Effect measure definition: Is the denominator all births, all CS, all women eligible for Robson grouping, or a subset (facility births only)?”

**Author response:** We have explicitly defined the effect measure as the proportion of primary cesarean sections, with the denominator being all births in all Robson Groups 1-10 (facility-based births only, as all included studies were institution-based). (See lines 88-98, 135-136, 159)

3. **Reviewer comment:** “Meta-analytic method: Specify the random-effects estimator used and whether proportions were transformed (logit, Freeman-Tukey double arcsine) to stabilize variance, this is particularly important for pooling prevalence/rates.”

**Author response:** We have specified the use of logit transformation and the DerSimonian-Laird estimator. (See page 7, lines 136-39)

4. **Reviewer comment:** “Please include planned/implemented strategies such as subgroup analysis (eg region, country income grouping, facility level, study design, time period, risk of bias)”

**Author response:** We have listed all pre-specified subgroup analyses by study period, SSA geographic region, country income level, facility type and level, CS cost-exemption policy, and JBI quality score (risk of bias). (See page 7, lines 143-45).

5. **Reviewer comment** “Sensitivity analyses: State whether analyses were repeated excluding high risk of bias studies, outliers, or small studies.”

**Author response:** We have described sensitivity analyses including leave-one-out influence analysis as well as restriction by study quality. (See page 7, lines 146-7).

**Methods**:

6. **Reviewer comment**: “A central concern is that primary studies may not record Robson criteria in comparable ways… Please explicitly describe: Robson mapping/harmonization: How did the authors ensure that women were assigned consistently to Groups 1-10 across studies? Were groups taken as reported by studies, or did the review team reconstruct grouping from raw obstetric variables…”

**Author response:** Robson groups were extracted exactly as reported in the original studies; no reconstruction from raw obstetric variables was performed. Only studies that explicitly applied the standard WHO Robson Ten-Group Classification System were included. See page 6, lines 104-111 (new subsection “Robson group classification and data harmonization”).

7. **Reviewer comment:** “Eligibility for inclusion per group: How were studies treated if they reported only some Robson groups, or if groups were aggregated (e.g., “1+2”)?”

**Author response:** Studies that reported only some Robson groups or aggregated groups (e.g., “1+2”) were not found and would be excluded. Only studies reporting complete data for all ten groups were eligible. (See page 4, lines 76-81, 104-111)

8. **Reviewer comment:** “Missing/unclear criteria: Was any imputation done? Were studies excluded or flagged when Robson criteria could not be verified?”

**Author response:** No imputation was performed. Studies with incomplete, inconsistent, or unverifiable Robson classification data were excluded. (See pages 6, lines 111, 104-106).

9. **Reviewer comment**: “Primary CS definition across studies: Many studies vary in defining primary CS… Please state how you accepted/standardized definitions and how this variability may bias pooling.”

**Author response:** We operationalized Primary CS as cesarean deliveries in women without a previous cesarean section (corresponding to Robson Groups 1, 2, 3, 4, and 6). It was not defined in the original studies. Groups 7-10 were excluded for conceptual clarity and comparability. We used this strict operational definition. (See page 4, lines 88-96 ).

**Discussion**

10. **Reviewer comment:** “The Discussion interprets high primary CS in Groups 1 and 2 as possible overuse/gaps. This may be correct, but interpretation depends strongly on risk profile. Please address: Across included studies, was there consistent reporting of maternal comorbidities and obstetric complications (e.g., hypertensive disorders, diabetes, HIV, placenta previa/accreta, fetal growth restriction, suspected macrosomia, chorioamnionitis, previous uterine surgery, anemia)?”

**Author response:** We have added an explicit statement that reporting of maternal comorbidities and obstetric complications was sparse and highly inconsistent across included studies. (See page 14, lines 235-249).

11. **Reviewer comment**: “If comorbidity data were sparse or inconsistent, state this clearly as a limitation: inability to adjust CS rates for risk differences could inflate or mask “overuse” signals.”

**Author response**: We have stated this clearly as a limitation and noted that the inability to adjust for risk differences may inflate apparent overuse signals in low-risk Robson groups. (See page 14, lines 250-254, 350-2).

12. **Reviewer comment**: “Consider whether certain comorbidities are unevenly distributed across regions/facility types and could partially explain heterogeneity and higher CS rates in low-risk groups (e.g., referral hospitals receiving complicated cases categorized within Robson groups based on basic criteria).”

**Author response:** We have added discussion of how referral hospitals (the majority of included studies) disproportionately receive complicated cases, which could elevate PCS rates even within nominally low-risk Robson groups and contribute to heterogeneity. (See page 14, lines 251-4).

13. **Reviewer comment**: “Given SSA’s diversity, differences in labor management and guidelines likely contribute to both CS rates and heterogeneity. Please discuss: Whether included studies spanned different health system levels (primary centers vs tertiary referral hospitals) and whether treatment/induction practices, fetal monitoring availability, partograph use, VBAC policies, and thresholds for “failure to progress” differ by region/country.”

**Author response:** We have added a dedicated paragraph addressing differences in health-system levels (primary/secondary vs tertiary referral hospitals), induction practices, fetal monitoring availability, partograph utilization, VBAC policies, and diagnostic thresholds for “failure to progress” or fetal distress across SSA, and their contribution to heterogeneity. (See page 16, lines 289-304).

14. **Reviewer comment**: “Results & data availability: make the raw extracted dataset and analysis code available. PLOS ONE strongly emphasizes transparency”

**Author response:** Thank you for this important suggestion. We have now made all the relevant data publicly available in Zenodo (<https://doi.org/10.5281/zenodo.19644772>), including the extracted dataset (CSV), cleaned Stata dataset (.dta), and Stata do-file used for analysis. (see page 129-130)

**Minor comments and editorial suggestions**

15. **Reviewer comment:** “Terminology: Clarify whether “prelabor CS” corresponds to Robson-defined onset of labor criteria and how elective/prelabor CS was identified in original studies.”

**Author response:** We have added explicit clarification that “prelabor CS” corresponds to Robson subcategories 2B and 4B (cesarean before labor) as defined by the World Health Organization. (See page 5, lines 101-102, 192).

All other minor editorial points have been addressed. Some citations were added as needed.

We believe these revisions satisfactorily resolve the concerns raised and have markedly improved the manuscript. We look forward to your feedback.

Sincerely,

Kindu Yinges Wondie (corresponding author)

On behalf of all co-authors

University of Gondar, Gondar, Ethiopia

Email: [kinduyinges2010@gmail.com](mailto:kinduyinges2010@gmail.com)
