# Supplementary material for: Primary cesarean section in Sub-Saharan Africa: A systematic review and meta-analysis using the Robson Ten-Group Classification System
Source: PLoS One. 2026 Jul 30;21(7):e0354911. doi: 10.1371/journal.pone.0354911 (PMC13422873; doi:10.1371/journal.pone.0354911)
Supplement: Attachment — Submitted filename: Comments for Authors and Editor.docx [file pone.0354911.s006.docx]

**Comments for Authors and Editor**

**Major Methodological Comments**

**#1.** The review is presented as a systematic review and meta-analysis - rate estimates, but the methodological framework for prevalence reviews is not adequately described. There is limited reference to established methodological guidance for prevalence meta-analysis e.g.- PERSyst guidance, particularly regarding handling of heterogeneity, transformation methods, pooling of proportions, and assessment of publication bias.

**#2.** Publication bias assessment using funnel plots and Egger’s test may not be appropriate for prevalence meta-analysis. The manuscript uses funnel plots and Egger’s test. - For prevalence studies with substantial heterogeneity (I² = 99.64% as reported in the study) and proportional data, these methods are very unreliable. Please consider using Doi plots and the LFK index, which are more suitable for prevalence meta-analyses.

**#3.** The quality assessment section contains inconsistencies regarding the appraisal tool used. The manuscript refers to the “JBI checklist for observational studies,” but the cited reference corresponds to methodological guidance for prevalence studies.
Please specify the exact name of the appraisal tool used (e.g., “Joanna Briggs Institute Critical Appraisal Checklist for Studies Reporting Prevalence Data”) and provide the appropriate citation.

**#4.** The method used to categorize study quality into low, medium, and high quality appears arbitrary and should be justified. The manuscript categorizes studies using cut-offs of 0–4, 5–7, and 8–9. Please cite appropriate reference and also please clarify whether these thresholds were pre-specified in the protocol or developed post hoc. If they were not protocol-defined, this should be acknowledged as a methodological deviation.

**#5.** The approach used for resolving disagreements in quality assessment may not be methodologically appropriate. The manuscript states that discrepancies were resolved “by averaging the scores.” Typically, disagreements in critical appraisal are resolved through discussion or involvement of a third reviewer rather than averaging numerical scores. Please justify this approach or revise accordingly.

**#6.** The rationale for pooling highly heterogeneous institution-based studies across SSA requires stronger justification. All included studies were institution-based cross-sectional studies from varying settings and healthcare systems. Pooling such heterogeneous data may limit interpretability and generalizability. The manuscript should better justify the appropriateness of pooled regional estimates and discuss contextual variability more explicitly.

**#7.** The manuscript should clarify whether the pooled estimate represents population-level or facility-level cesarean section patterns. Since all included studies are institution-based and many appear tertiary/referral-center based, the pooled estimate likely reflects facility-level patterns rather than regional population prevalence. This distinction should be emphasized throughout the manuscript, particularly in the abstract, discussion, and conclusions.

**#8.** Some interpretations regarding “overuse” of cesarean section appear overly assertive given the nature of the included data. The manuscript repeatedly interprets elevated PCS rates as evidence of “overuse.”
However, many included studies are from tertiary referral hospitals managing high-risk obstetric populations. Without detailed indication-level data, caution is needed when attributing findings to inappropriate or unnecessary cesarean use.
