# Supplementary material for: Primary cesarean section in Sub-Saharan Africa: A systematic review and meta-analysis using the Robson Ten-Group Classification System
Source: PLoS One. 2026 Jul 30;21(7):e0354911. doi: 10.1371/journal.pone.0354911 (PMC13422873; doi:10.1371/journal.pone.0354911)
Supplement: Attachment — Submitted filename: Response_to_Reviewers_auresp_2.docx [file pone.0354911.s008.docx]

**Response to Reviewers**

**Manuscript ID:** PONE-D-25-55783R1

**Title:** Primary cesarean section in sub-Saharan Africa: a systematic review and meta-analysis using the Robson Ten-Group Classification System

**Dear Academic Editor and Reviewers**,

We thank the editors and reviewers for their continued engagement with our manuscript. We have carefully addressed all remaining concerns and the major methodological critiques raised. Below we provide a point-by-point response.

**Reviewer #1**

**#1**. The issues identified in the first review round have been adequately resolved, and the manuscript is now largely suitable for publication. One remaining point should be checked carefully before acceptance. In the abstract, introduction, and discussion, particularly where references 1, 2, 8, and 9 are cited, the authors appear to interpret the findings as indicating that overuse of caesarean section directly leads to increased perinatal mortality. However, the cited studies do not establish a causal relationship between caesarean section itself and worse neonatal outcomes.
The relevant passages should therefore be reviewed and, if necessary, revised to ensure that the interpretation accurately reflects the cited literature. The wording should make clear whether poorer neonatal outcomes are associated with the circumstances and management preceding caesarean section, including delayed or non-timely intervention, rather than implying that caesarean section itself is the causal factor. After this clarification, I consider the manuscript acceptable.

**Response:** We agree with the reviewer and have revised the wording throughout the manuscript to avoid implying causality.

\#**2**. Minor issue, formatting in line 48 WHO ().

Response: corrected.

**Reviewer #2 (Major Methodological Comments)**

**#1.** The review is presented as a systematic review and meta-analysis - rate estimates, but the methodological framework for prevalence reviews is not adequately described. There is limited reference to established methodological guidance for prevalence meta-analysis e.g.- PERSyst guidance, particularly regarding handling of heterogeneity, transformation methods, pooling of proportions, and assessment of publication bias.

**Response:** We thank the reviewer for this important methodological suggestion. We have revised the Methods section to explicitly align the review with established guidance for prevalence meta-analysis, including the Joanna Briggs Institute methodology and the PERSyst guidance. We now clarify that proportions were logit-transformed prior to pooling (Barendregt et al., 2013) to stabilize variances, with results back-transformed for interpretation. We also provide a clearer description of heterogeneity assessment using Cochran’s Q and I² statistics with predefined thresholds. In addition, we have strengthened the description of our approach to handling heterogeneity and publication bias to improve transparency and methodological rigor.

**#2.** Publication bias assessment using funnel plots and Egger’s test may not be appropriate for prevalence meta-analysis. The manuscript uses funnel plots and Egger’s test. - For prevalence studies with substantial heterogeneity (I² = 99.64% as reported in the study) and proportional data, these methods are very unreliable. Please consider using Doi plots and the LFK index, which are more suitable for prevalence meta-analyses.

**Response:** Thank you, dear reviewer. We have now conducted a Doi plot with LFK index as recommended for prevalence meta-analyses. The LFK index was -0.27, indicating no significant small-study effects. The Doi plot now appears as Figure 4 in the main manuscript. In the revised manuscript, we report the LFK index value and interpret symmetry accordingly. The funnel plot has been removed from the main results.

**#3.** The quality assessment section contains inconsistencies regarding the appraisal tool used. The manuscript refers to the “JBI checklist for observational studies,” but the cited reference corresponds to methodological guidance for prevalence studies. Please specify the exact name of the appraisal tool used (e.g., “Joanna Briggs Institute Critical Appraisal Checklist for Studies Reporting Prevalence Data”) and provide the appropriate citation.

**Response:** Thank you for your insightful comment. We have corrected this. We now cite the Joanna Briggs Institute Critical Appraisal Checklist for Studies Reporting Prevalence Data specifically, with the appropriate reference. We now cite the checklist correctly and provide the full reference. The checklist itself is included as Supplementary File S3.

**#4.** The method used to categorize study quality into low, medium, and high quality appears arbitrary and should be justified. The manuscript categorizes studies using cut-offs of 0–4, 5–7, and 8–9. Please cite appropriate reference and also please clarify whether these thresholds were pre-specified in the protocol or developed post hoc. If they were not protocol-defined, this should be acknowledged as a methodological deviation.

**Response:** We thank the reviewer for this important observation. We would like to clarify that the study protocol did not pre-specify any JBI score thresholds for categorizing studies into low-, medium-, or high-quality groups (0–4, 5–7, and 8–9). The protocol specified only that the Joanna Briggs Institute (JBI) Critical Appraisal Checklist for Studies Reporting Prevalence Data would be used to assess methodological quality and risk of bias. We acknowledge that the quality categories (0–4, 5–7, and 8–9) were derived post hoc from the 0–9 JBI scoring range and were used solely for subgroup analysis. We also identified an error in the original manuscript stating that “only studies scoring ≥5 (medium or high quality) were included.” This statement was incorrect. No studies were excluded based on their JBI quality scores, and all eligible studies were retained in the review regardless of their appraisal results. In fact, all included studies scored between 7 and 9 on the JBI checklist (21 studies scored 8–9, and 4 studies scored 7) (**Supplementary file 3**). We have revised the manuscript by removing the low-, medium-, and high-quality classifications, as well as the statement indicating that studies scoring ≥5 were included. We now clarify that JBI appraisal was conducted to assess methodological quality and risk of bias.

**#5.** The approach used for resolving disagreements in quality assessment may not be methodologically appropriate. The manuscript states that discrepancies were resolved “by averaging the scores.” Typically, disagreements in critical appraisal are resolved through discussion or involvement of a third reviewer rather than averaging numerical scores. Please justify this approach or revise accordingly.

**Response:** We thank the reviewer for this important observation and agree that averaging quality assessment scores is not the preferred approach for resolving discrepancies in critical appraisal. We have therefore revised the manuscript accordingly. In the revised manuscript, disagreements between reviewers during the quality assessment process were resolved through discussion and consensus. Where consensus could not be reached, a third reviewer (senior author) made the final decision. The statement indicating that discrepancies were resolved by averaging scores has been removed.

**#6.** The rationale for pooling highly heterogeneous institution-based studies across SSA requires stronger justification. All included studies were institution-based cross-sectional studies from varying settings and healthcare systems. Pooling such heterogeneous data may limit interpretability and generalizability. The manuscript should better justify the appropriateness of pooled regional estimates and discuss contextual variability more explicitly.

**Response:** We thank the reviewer for this important observation. We agree that substantial clinical and methodological heterogeneity exists across institution-based studies conducted in different healthcare settings within sub-Saharan Africa. However, all included studies applied the Robson Ten-Group Classification System, which provides a standardized and internationally recognized framework for assessing cesarean section practices. This common classification enabled the synthesis of primary cesarean section rates across studies despite differences in healthcare systems and facility characteristics. To address this concern, we have strengthened the justification for pooling in the manuscript. Specifically, we now explain that a random-effects model was used to account for between-study variability and that the pooled estimate should be interpreted as a regional facility-based summary rather than a population-level prevalence estimate. We have also expanded the Discussion to more explicitly acknowledge contextual variability across settings, including differences in referral patterns, facility level, case mix, healthcare resources, and clinical practices. In addition, we conducted univariable meta-regression analyses using study period, geographic region, country income category, institution type, facility scope, and cesarean section cost-exemption policy to further explore sources of heterogeneity. None of these study-level characteristics significantly explained the observed between-study variability, suggesting that heterogeneity is likely driven by multiple interacting clinical and health-system factors not captured in the available data.

**#7.** The manuscript should clarify whether the pooled estimate represents population-level or facility-level cesarean section patterns. Since all included studies are institution-based and many appear tertiary/referral-center based, the pooled estimate likely reflects facility-level patterns rather than regional population prevalence. This distinction should be emphasized throughout the manuscript, particularly in the abstract, discussion, and conclusions.

**Response:** Thank you. We have revised the manuscript throughout to clearly state that the pooled primary cesarean section rate reflects facility-based births only. This is now emphasized in the abstract, methods, results, discussion, and conclusion. A new sentence in the limitations explicitly states that the estimates do not represent population-level prevalence due to underrepresentation of home births and primary-level facilities.

**#8.** Some interpretations regarding “overuse” of cesarean section appear overly assertive given the nature of the included data. The manuscript repeatedly interprets elevated PCS rates as evidence of “overuse.”
However, many included studies are from tertiary referral hospitals managing high-risk obstetric populations. Without detailed indication-level data, caution is needed when attributing findings to inappropriate or unnecessary cesarean use.

**Response:** We agree with the reviewer. We have systematically softened language throughout the manuscript, replacing “overuse” with “potential overuse,” “possible overuse,” “may indicate overuse,” or “raises concern for overuse.” We also added explicit cautions that without detailed indication data, definitive attribution to overuse is not possible. Reviewer #1 also raised a related point about causal language (CS → mortality), which we have addressed.

**Additional editorial changes:**

- All figures have been updated where indicated.
- The data availability statement remains correct and the Zenodo repository is active.
- The reference list has been checked for consistency.

We believe the manuscript is now methodologically sound and appropriately cautious in its interpretations. We respectfully request that the manuscript be accepted for publication.

Sincerely,

Kindu Yinges Wondie (corresponding author)

On behalf of all co-authors
