# Supplementary material for: Primary cesarean section in Sub-Saharan Africa: A systematic review and meta-analysis using the Robson Ten-Group Classification System
Source: PLoS One. 2026 Jul 30;21(7):e0354911. doi: 10.1371/journal.pone.0354911 (PMC13422873; doi:10.1371/journal.pone.0354911)
Supplement: Peer review history

# Peer review: Decision Letter 0

**DOI:** 10.1371/journal.pone.0354911.r001

**Authors:** Emma Campbell

9 Apr 2026

PONE-D-25-55783

Primary cesarean section rate in sub-Saharan Africa: a systematic review and meta-analysis using the Robson Ten-Group Classification System

PLOS One

Dear Dr. Wondie,

Thank you for submitting your manuscript to PLOS ONE. After careful consideration, we feel that it has merit but does not fully meet PLOS ONE’s publication criteria as it currently stands. Therefore, we invite you to submit a revised version of the manuscript that addresses the points raised during the review process.

Please submit your revised manuscript by May 23 2026 11:59PM. If you will need more time than this to complete your revisions, please reply to this message or contact the journal office at plosone@plos.org. When you're ready to submit your revision, log on to https://www.editorialmanager.com/pone/ and select the 'Submissions Needing Revision' folder to locate your manuscript file.

Please include the following items when submitting your revised manuscript:

- A letter that responds to each point raised by the academic editor and reviewer(s). You should upload this letter as a separate file labeled 'Response to Reviewers'.
- A marked-up copy of your manuscript that highlights changes made to the original version. You should upload this as a separate file labeled 'Revised Manuscript with Track Changes'.
- An unmarked version of your revised paper without tracked changes. You should upload this as a separate file labeled 'Manuscript'.

If you would like to make changes to your financial disclosure, please include your updated statement in your cover letter. Guidelines for resubmitting your figure files are available below the reviewer comments at the end of this letter.

If applicable, we recommend that you deposit your laboratory protocols in protocols.io to enhance the reproducibility of your results. Protocols.io assigns your protocol its own identifier (DOI) so that it can be cited independently in the future. For instructions see: https://journals.plos.org/plosone/s/submission-guidelines#loc-laboratory-protocols. Additionally, PLOS ONE offers an option for publishing peer-reviewed Lab Protocol articles, which describe protocols hosted on protocols.io. Read more information on sharing protocols at https://plos.org/protocols?utm_medium=editorial-email&utm_source=authorletters&utm_campaign=protocols.

As the corresponding author, your ORCID iD is verified in the submission system and will appear in the published article. PLOS supports the use of ORCID, and we encourage all coauthors to register for an ORCID iD and use it as well. Please encourage your coauthors to verify their ORCID iD within the submission system before final acceptance, as unverified ORCID iDs will not appear in the published article. *Only*  the individual author can complete the verification step; PLOS staff *cannot*  verify ORCID iDs on behalf of authors.

We look forward to receiving your revised manuscript.

Kind regards,

Emma Campbell, Ph.D

Staff Editor

PLOS One

Journal Requirements:

When submitting your revision, we need you to address these additional requirements.

1. Please ensure that your manuscript meets PLOS ONE's style requirements, including those for file naming. The PLOS ONE style templates can be found at

https://journals.plos.org/plosone/s/file?id=wjVg/PLOSOne_formatting_sample_main_body.pdf and

https://journals.plos.org/plosone/s/file?id=ba62/PLOSOne_formatting_sample_title_authors_affiliations.pdf

2. If the reviewer comments include a recommendation to cite specific previously published works, please review and evaluate these publications to determine whether they are relevant and should be cited. There is no requirement to cite these works unless the editor has indicated otherwise.

**Additional Editor Comments:**

Please note that we have only been able to secure a single reviewer to assess your manuscript. We are issuing a decision on your manuscript at this point to prevent further delays in the evaluation of your manuscript. Please be aware that the editor who handles your revised manuscript might find it necessary to invite additional reviewers to assess this work once the revised manuscript is submitted. However, we will aim to proceed on the basis of this single review if possible.

-->The reviewer comments are available below. Please review the comments and revise the manuscript accordingly.

[Note: HTML markup is below. Please do not edit.]

Reviewers' comments:

Reviewer's Responses to Questions

**Comments to the Author**

1. Is the manuscript technically sound, and do the data support the conclusions?

The manuscript must describe a technically sound piece of scientific research with data that supports the conclusions. Experiments must have been conducted rigorously, with appropriate controls, replication, and sample sizes. The conclusions must be drawn appropriately based on the data presented.

Reviewer #1: Yes

**********

2. Has the statistical analysis been performed appropriately and rigorously?

Reviewer #1: Yes

**********

3. Have the authors made all data underlying the findings in their manuscript fully available?

The PLOS Data policy requires authors to make all data underlying the findings described in their manuscript fully available without restriction, with rare exception (please refer to the Data Availability Statement in the manuscript PDF file). The data should be provided as part of the manuscript or its supporting information, or deposited to a public repository. For example, in addition to summary statistics, the data points behind means, medians and variance measures should be available. If there are restrictions on publicly sharing data—e.g. participant privacy or use of data from a third party—those must be specified.

Reviewer #1: No

**********

4. Is the manuscript presented in an intelligible fashion and written in standard English?

PLOS ONE does not copyedit accepted manuscripts, so the language in submitted articles must be clear, correct, and unambiguous. Any typographical or grammatical errors should be corrected at revision, so please note any specific errors here.

Reviewer #1: Yes

**********

5. Review Comments to the Author

Please use the space provided to explain your answers to the questions above. You may also include additional comments for the author, including concerns about dual publication, research ethics, or publication ethics. (Please upload your review as an attachment if it exceeds 20,000 characters)

Reviewer #1: Summary

This manuscript addresses an important and policy-relevant topic: primary cesarean section (CS) rates in sub-Saharan Africa (SSA) using the Robson Ten-Group Classification System. The study is timely and potentially impactful given ongoing concern about CS overuse and variable obstetric capacity across SSA. and globally The study generally meets scientific criteria for publication, but several elements require strengthening to meet PLOS ONE expectations for transparency, reproducibility, and careful interpretation of heterogeneity.

Major comments

Introduction: shorten and sharpen the rationale

The Introduction is generally clear but could be more concise, ie reduce narrative text; keep 2–3 short paragraphs ending with a precise objective and (ideally) a brief statement of the analytical approach

Methods: pooled data approach must be more explicit and reproducible

While databases, timeframe, software, and random-effects model are described, offer a greater detail to allow replication.

e.g. clarify:

Effect measure definition: Is the denominator all births, all CS, all women eligible for Robson grouping, or a subset (facility births only)?

Meta-analytic method: Specify the random-effects estimator used and whether proportions were transformed (logit, Freeman–Tukey double arcsine) to stabilize variance—this is particularly important for pooling prevalence/rates.

Please include planned/implemented strategies such as subgroup analysis (eg region, country income grouping, facility level, study design, time period, risk of bias)

Sensitivity analyses: State whether analyses were repeated excluding high risk of bias studies, outliers, or small studies.

These additions are necessary for readers to assess the credibility of the pooled estimate.

Methods: explain how different sources handled Robson data recorded differently

A central concern is that primary studies may not record Robson criteria in comparable ways, and some may apply modified versions or have incomplete reporting (e.g., parity, previous CS, gestational age, onset of labor, fetal presentation, plurality).

Please explicitly describe:

Robson mapping/harmonization: How did the authors ensure that women were assigned consistently to Groups 1–10 across studies? Were groups taken as reported by studies, or did the review team reconstruct grouping from raw obstetric variables (mostly unlikely in literature-based meta-analysis)?

Eligibility for inclusion per group: How were studies treated if they reported only some Robson groups, or if groups were aggregated (e.g., “1+2”)?

Missing/unclear criteria: Was any imputation done? Were studies excluded or flagged when Robson criteria could not be verified?

Primary CS definition across studies: Many studies vary in defining primary CS (first-ever CS, or no prior uterine scar, or no previous CS but possibly prior uterine surgery). Please state how you accepted/standardized definitions and how this variability may bias pooling.

Discussion: address availability of comorbidity data that could influence Robson rates

The Discussion interprets high primary CS in Groups 1 and 2 as possible overuse/gaps. This may be correct, but interpretation depends strongly on risk profile.

Please address:

Across included studies, was there consistent reporting of maternal comorbidities and obstetric complications (e.g., hypertensive disorders, diabetes, HIV, placenta previa/accreta, fetal growth restriction, suspected macrosomia, chorioamnionitis, previous uterine surgery, anemia)?

If comorbidity data were sparse or inconsistent, state this clearly as a limitation: inability to adjust CS rates for risk differences could inflate or mask “overuse” signals.

Consider whether certain comorbidities are unevenly distributed across regions/facility types and could partially explain heterogeneity and higher CS rates in low-risk groups (e.g., referral hospitals receiving complicated cases categorized within Robson groups based on basic criteria).

Discussion: regional differences in treatment guidelines and practice patterns

Given SSA’s diversity, differences in labor management and guidelines likely contribute to both CS rates and heterogeneity.

Please discuss:

Whether included studies spanned different health system levels (primary centers vs tertiary referral hospitals) and whether treatment/induction practices, fetal monitoring availability, partograph use, VBAC policies, and thresholds for “failure to progress” differ by region/country.

Results & data availability: make the raw extracted dataset and analysis code available

PLOS ONE strongly emphasizes transparency

Minor comments and editorial suggestions

Terminology: Clarify whether “prelabor CS” corresponds to Robson-defined onset of labor criteria and how elective/prelabor CS was identified in original studies.

Overall assessment

The manuscript addresses an important SSA maternal health issue and uses an internationally accepted classification framework. The results are plausible and potentially useful for audit and policy. With a shortened Introduction, clearer and more transparent Methods (especially around pooling and harmonizing Robson criteria), and a strengthened Discussion on comorbidities and regional guideline/practice variation the paper would meet PLOS ONE’s standards and be suitable for publication.

**********

6. PLOS authors have the option to publish the peer review history of their article (what does this mean?). If published, this will include your full peer review and any attached files.

If you choose “no”, your identity will remain anonymous but your review may still be made public.

**Do you want your identity to be public for this peer review?**  For information about this choice, including consent withdrawal, please see our Privacy Policy.

Reviewer #1: No

**********

[NOTE: If reviewer comments were submitted as an attachment file, they will be attached to this email and accessible via the submission site. Please log into your account, locate the manuscript record, and check for the action link "View Attachments". If this link does not appear, there are no attachment files.]

To ensure your figures meet our technical requirements, please review our figure guidelines: https://journals.plos.org/plosone/s/figures

You may also use PLOS’s free figure tool, NAAS, to help you prepare publication quality figures: https://journals.plos.org/plosone/s/figures#loc-tools-for-figure-preparation.

NAAS will assess whether your figures meet our technical requirements by comparing each figure against our figure specifications.

# Author comment: Author response to Decision Letter 1

**DOI:** 10.1371/journal.pone.0354911.r002

18 Apr 2026

Response to Reviewers

PONE-D-25-55783

Primary cesarean section rate in sub-Saharan Africa: a systematic review and meta-analysis using the Robson Ten-Group Classification System

Dear Reviewer #1 and Editor,

We sincerely thank Reviewer #1 for the thorough, constructive, and highly detailed comments, which have substantially improved the transparency, reproducibility, and interpretive rigor of the manuscript. Below we address each point exactly as raised. Where the reviewer combined several related issues into one comment, we have broken them down into specific sub-points for precision.

Major comments

Introduction

1. Reviewer comment: “The Introduction is generally clear but could be more concise, ie reduce narrative text; keep 2-3 short paragraphs ending with a precise objective and (ideally) a brief statement of the analytical approach”

Author response: The introduction has been shortened and restructured. The final paragraph now clearly states the study objective and the analytical approach using the Robson Ten-Group Classification System for pooled estimation of primary cesarean section rates. (See page 3, lines 42-62).

Methods

2. Reviewer comment:

“While databases, timeframe, software, and random-effects model are described, offer a greater detail to allow replication. e.g. clarify: Effect measure definition: Is the denominator all births, all CS, all women eligible for Robson grouping, or a subset (facility births only)?”

Author response: We have explicitly defined the effect measure as the proportion of primary cesarean sections, with the denominator being all births in all Robson Groups 1-10 (facility-based births only, as all included studies were institution-based). (See lines 88-98, 135-136, 159)

3. Reviewer comment: “Meta-analytic method: Specify the random-effects estimator used and whether proportions were transformed (logit, Freeman-Tukey double arcsine) to stabilize variance, this is particularly important for pooling prevalence/rates.”

Author response: We have specified the use of logit transformation and the DerSimonian-Laird estimator. (See page 7, lines 136-39)

4. Reviewer comment: “Please include planned/implemented strategies such as subgroup analysis (eg region, country income grouping, facility level, study design, time period, risk of bias)”

Author response: We have listed all pre-specified subgroup analyses by study period, SSA geographic region, country income level, facility type and level, CS cost-exemption policy, and JBI quality score (risk of bias). (See page 7, lines 143-45).

5. Reviewer comment “Sensitivity analyses: State whether analyses were repeated excluding high risk of bias studies, outliers, or small studies.”

Author response: We have described sensitivity analyses including leave-one-out influence analysis as well as restriction by study quality. (See page 7, lines 146-7).

Methods:

6. Reviewer comment: “A central concern is that primary studies may not record Robson criteria in comparable ways… Please explicitly describe: Robson mapping/harmonization: How did the authors ensure that women were assigned consistently to Groups 1-10 across studies? Were groups taken as reported by studies, or did the review team reconstruct grouping from raw obstetric variables…”

Author response: Robson groups were extracted exactly as reported in the original studies; no reconstruction from raw obstetric variables was performed. Only studies that explicitly applied the standard WHO Robson Ten-Group Classification System were included. See page 6, lines 104-111 (new subsection “Robson group classification and data harmonization”).

7. Reviewer comment: “Eligibility for inclusion per group: How were studies treated if they reported only some Robson groups, or if groups were aggregated (e.g., “1+2”)?”

Author response: Studies that reported only some Robson groups or aggregated groups (e.g., “1+2”) were not found and would be excluded. Only studies reporting complete data for all ten groups were eligible. (See page 4, lines 76-81, 104-111)

8. Reviewer comment: “Missing/unclear criteria: Was any imputation done? Were studies excluded or flagged when Robson criteria could not be verified?”

Author response: No imputation was performed. Studies with incomplete, inconsistent, or unverifiable Robson classification data were excluded. (See pages 6, lines 111, 104-106).

9. Reviewer comment: “Primary CS definition across studies: Many studies vary in defining primary CS… Please state how you accepted/standardized definitions and how this variability may bias pooling.”

Author response: We operationalized Primary CS as cesarean deliveries in women without a previous cesarean section (corresponding to Robson Groups 1, 2, 3, 4, and 6). It was not defined in the original studies. Groups 7-10 were excluded for conceptual clarity and comparability. We used this strict operational definition. (See page 4, lines 88-96 ).

Discussion

10. Reviewer comment: “The Discussion interprets high primary CS in Groups 1 and 2 as possible overuse/gaps. This may be correct, but interpretation depends strongly on risk profile. Please address: Across included studies, was there consistent reporting of maternal comorbidities and obstetric complications (e.g., hypertensive disorders, diabetes, HIV, placenta previa/accreta, fetal growth restriction, suspected macrosomia, chorioamnionitis, previous uterine surgery, anemia)?”

Author response: We have added an explicit statement that reporting of maternal comorbidities and obstetric complications was sparse and highly inconsistent across included studies. (See page 14, lines 235-249).

11. Reviewer comment: “If comorbidity data were sparse or inconsistent, state this clearly as a limitation: inability to adjust CS rates for risk differences could inflate or mask “overuse” signals.”

Author response: We have stated this clearly as a limitation and noted that the inability to adjust for risk differences may inflate apparent overuse signals in low-risk Robson groups. (See page 14, lines 250-254, 350-2).

12. Reviewer comment: “Consider whether certain comorbidities are unevenly distributed across regions/facility types and could partially explain heterogeneity and higher CS rates in low-risk groups (e.g., referral hospitals receiving complicated cases categorized within Robson groups based on basic criteria).”

Author response: We have added discussion of how referral hospitals (the majority of included studies) disproportionately receive complicated cases, which could elevate PCS rates even within nominally low-risk Robson groups and contribute to heterogeneity. (See page 14, lines 251-4).

13. Reviewer comment: “Given SSA’s diversity, differences in labor management and guidelines likely contribute to both CS rates and heterogeneity. Please discuss: Whether included studies spanned different health system levels (primary centers vs tertiary referral hospitals) and whether treatment/induction practices, fetal monitoring availability, partograph use, VBAC policies, and thresholds for “failure to progress” differ by region/country.”

Author response: We have added a dedicated paragraph addressing differences in health-system levels (primary/secondary vs tertiary referral hospitals), induction practices, fetal monitoring availability, partograph utilization, VBAC policies, and diagnostic thresholds for “failure to progress” or fetal distress across SSA, and their contribution to heterogeneity. (See page 16, lines 289-304).

14. Reviewer comment: “Results & data availability: make the raw extracted dataset and analysis code available. PLOS ONE strongly emphasizes transparency”

Author response: Thank you for this important suggestion. We have now made all the relevant data publicly available in Zenodo (https://doi.org/10.5281/zenodo.19644772), including the extracted dataset (CSV), cleaned Stata dataset (.dta), and Stata do-file used for analysis. (see page 129-130)

Minor comments and editorial suggestions

15. Reviewer comment: “Terminology: Clarify whether “prelabor CS” corresponds to Robson-defined onset of labor criteria and how elective/prelabor CS was identified in original studies.”

Author response: We have added explicit clarification that “prelabor CS” corresponds to Robson subcategories 2B and 4B (cesarean before labor) as defined by the World Health Organization. (See page 5, lines 101-102, 192).

All other minor editorial points have been addressed. Some citations were added as needed.

We believe these revisions satisfactorily resolve the concerns raised and have markedly improved the manuscript. We look forward to your feedback.

Sincerely,

Kindu Yinges Wondie (corresponding author)

On behalf of all co-authors

University of Gondar, Gondar, Ethiopia

Email: kinduyinges2010@gmail.com

Attachment Submitted filename: Response to Reviewers.docx

# Peer review: Decision Letter 1

**DOI:** 10.1371/journal.pone.0354911.r003

**Authors:** Pijush Kanti Khan

2 Jun 2026

PONE-D-25-55783R1

Primary cesarean section in sub-Saharan Africa: a systematic review and meta-analysis using the Robson Ten-Group Classification System

PLOS One

Dear Dr. Wondie,

Thank you for submitting your manuscript to PLOS ONE. After careful consideration, we feel that it has merit but does not fully meet PLOS ONE’s publication criteria as it currently stands. Therefore, we invite you to submit a revised version of the manuscript that addresses the points raised during the review process.

Please submit your revised manuscript by Jul 17 2026 11:59PM. If you will need more time than this to complete your revisions, please reply to this message or contact the journal office at plosone@plos.org. When you're ready to submit your revision, log on to https://www.editorialmanager.com/pone/ and select the 'Submissions Needing Revision' folder to locate your manuscript file.

Please include the following items when submitting your revised manuscript:

- A letter that responds to each point raised by the academic editor and reviewer(s). You should upload this letter as a separate file labeled 'Response to Reviewers'.
- A marked-up copy of your manuscript that highlights changes made to the original version. You should upload this as a separate file labeled 'Revised Manuscript with Track Changes'.
- An unmarked version of your revised paper without tracked changes. You should upload this as a separate file labeled 'Manuscript'.

If you would like to make changes to your financial disclosure, please include your updated statement in your cover letter. Guidelines for resubmitting your figure files are available below the reviewer comments at the end of this letter.

If applicable, we recommend that you deposit your laboratory protocols in protocols.io to enhance the reproducibility of your results. Protocols.io assigns your protocol its own identifier (DOI) so that it can be cited independently in the future. For instructions see: https://journals.plos.org/plosone/s/submission-guidelines#loc-laboratory-protocols. Additionally, PLOS ONE offers an option for publishing peer-reviewed Lab Protocol articles, which describe protocols hosted on protocols.io. Read more information on sharing protocols at https://plos.org/protocols?utm_medium=editorial-email&utm_source=authorletters&utm_campaign=protocols.

As the corresponding author, your ORCID iD is verified in the submission system and will appear in the published article. PLOS supports the use of ORCID, and we encourage all coauthors to register for an ORCID iD and use it as well. Please encourage your coauthors to verify their ORCID iD within the submission system before final acceptance, as unverified ORCID iDs will not appear in the published article. *Only*  the individual author can complete the verification step; PLOS staff *cannot*  verify ORCID iDs on behalf of authors.

We look forward to receiving your revised manuscript.

Kind regards,

Pijush Kanti Khan, Ph.D.

Academic Editor

PLOS One

Journal Requirements:

If the reviewer comments include a recommendation to cite specific previously published works, please review and evaluate these publications to determine whether they are relevant and should be cited. There is no requirement to cite these works unless the editor has indicated otherwise.

[Note: HTML markup is below. Please do not edit.]

Reviewers' comments:

Reviewer's Responses to Questions

**Comments to the Author**

1. If the authors have adequately addressed your comments raised in a previous round of review and you feel that this manuscript is now acceptable for publication, you may indicate that here to bypass the “Comments to the Author” section, enter your conflict of interest statement in the “Confidential to Editor” section, and submit your "Accept" recommendation.

Reviewer #1: All comments have been addressed

Reviewer #2: (No Response)

**********

2. Is the manuscript technically sound, and do the data support the conclusions?

The manuscript must describe a technically sound piece of scientific research with data that supports the conclusions. Experiments must have been conducted rigorously, with appropriate controls, replication, and sample sizes. The conclusions must be drawn appropriately based on the data presented.

Reviewer #1: Yes

Reviewer #2: Partly

**********

3. Has the statistical analysis been performed appropriately and rigorously?

Reviewer #1: Yes

Reviewer #2: No

**********

4. Have the authors made all data underlying the findings in their manuscript fully available?

The PLOS Data policy requires authors to make all data underlying the findings described in their manuscript fully available without restriction, with rare exception (please refer to the Data Availability Statement in the manuscript PDF file). The data should be provided as part of the manuscript or its supporting information, or deposited to a public repository. For example, in addition to summary statistics, the data points behind means, medians and variance measures should be available. If there are restrictions on publicly sharing data—e.g. participant privacy or use of data from a third party—those must be specified.

Reviewer #1: Yes

Reviewer #2: Yes

**********

5. Is the manuscript presented in an intelligible fashion and written in standard English?

PLOS ONE does not copyedit accepted manuscripts, so the language in submitted articles must be clear, correct, and unambiguous. Any typographical or grammatical errors should be corrected at revision, so please note any specific errors here.

Reviewer #1: Yes

Reviewer #2: Yes

**********

6. Review Comments to the Author

Please use the space provided to explain your answers to the questions above. You may also include additional comments for the author, including concerns about dual publication, research ethics, or publication ethics. (Please upload your review as an attachment if it exceeds 20,000 characters)

Reviewer #1: The issues identified in the first review round have been adequately resolved, and the manuscript is now largely suitable for publication.

One remaining point should be checked carefully before acceptance. In the abstract, introduction, and discussion, particularly where references 1, 2, 8, and 9 are cited, the authors appear to interpret the findings as indicating that overuse of caesarean section directly leads to increased perinatal mortality. However, the cited studies do not establish a causal relationship between caesarean section itself and worse neonatal outcomes.

The relevant passages should therefore be reviewed and, if necessary, revised to ensure that the interpretation accurately reflects the cited literature. The wording should make clear whether poorer neonatal outcomes are associated with the circumstances and management preceding caesarean section, including delayed or non-timely intervention, rather than implying that caesarean section itself is the causal factor.

After this clarification, I consider the manuscript acceptable.

minor issue, formatting in line 48 WHO ()

Reviewer #2: Overall, the manuscript addresses an important and relevant public health issue; however, several major methodological concerns need further clarification and strengthening. In particular, the methodological framework for prevalence meta-analysis, handling of extreme heterogeneity, publication bias assessment, and quality appraisal procedures require greater justification and alignment with established guidance for prevalence reviews. Additionally, interpretations regarding cesarean section “overuse” should be presented more cautiously, considering that the pooled estimates are largely derived from institution-based and referral-center studies rather than population-level data.

**********

7. PLOS authors have the option to publish the peer review history of their article (what does this mean?). If published, this will include your full peer review and any attached files.

If you choose “no”, your identity will remain anonymous but your review may still be made public.

**Do you want your identity to be public for this peer review?**  For information about this choice, including consent withdrawal, please see our Privacy Policy.

Reviewer #1: No

Reviewer #2: **Yes:** Dr Anuj Kumar Pandey

**********

[NOTE: If reviewer comments were submitted as an attachment file, they will be attached to this email and accessible via the submission site. Please log into your account, locate the manuscript record, and check for the action link "View Attachments". If this link does not appear, there are no attachment files.]

To ensure your figures meet our technical requirements, please review our figure guidelines: https://journals.plos.org/plosone/s/figures

You may also use PLOS’s free figure tool, NAAS, to help you prepare publication quality figures: https://journals.plos.org/plosone/s/figures#loc-tools-for-figure-preparation.

NAAS will assess whether your figures meet our technical requirements by comparing each figure against our figure specifications.

Attachment Submitted filename: Comments for Authors and Editor.docx

# Author comment: Author response to Decision Letter 2

**DOI:** 10.1371/journal.pone.0354911.r004

19 Jun 2026

Response to Reviewers

Manuscript ID: PONE-D-25-55783R1

Title: Primary cesarean section in sub-Saharan Africa: a systematic review and meta-analysis using the Robson Ten-Group Classification System

Dear Academic Editor and Reviewers,

We thank the editors and reviewers for their continued engagement with our manuscript. We have carefully addressed all remaining concerns and the major methodological critiques raised. Below we provide a point-by-point response.

Reviewer #1

#1. The issues identified in the first review round have been adequately resolved, and the manuscript is now largely suitable for publication. One remaining point should be checked carefully before acceptance. In the abstract, introduction, and discussion, particularly where references 1, 2, 8, and 9 are cited, the authors appear to interpret the findings as indicating that overuse of caesarean section directly leads to increased perinatal mortality. However, the cited studies do not establish a causal relationship between caesarean section itself and worse neonatal outcomes.

The relevant passages should therefore be reviewed and, if necessary, revised to ensure that the interpretation accurately reflects the cited literature. The wording should make clear whether poorer neonatal outcomes are associated with the circumstances and management preceding caesarean section, including delayed or non-timely intervention, rather than implying that caesarean section itself is the causal factor. After this clarification, I consider the manuscript acceptable.

Response: We agree with the reviewer and have revised the wording throughout the manuscript to avoid implying causality.

#2. Minor issue, formatting in line 48 WHO ().

Response: corrected.

Reviewer #2 (Major Methodological Comments)

#1. The review is presented as a systematic review and meta-analysis - rate estimates, but the methodological framework for prevalence reviews is not adequately described. There is limited reference to established methodological guidance for prevalence meta-analysis e.g.- PERSyst guidance, particularly regarding handling of heterogeneity, transformation methods, pooling of proportions, and assessment of publication bias.

Response: We thank the reviewer for this important methodological suggestion. We have revised the Methods section to explicitly align the review with established guidance for prevalence meta-analysis, including the Joanna Briggs Institute methodology and the PERSyst guidance. We now clarify that proportions were logit-transformed prior to pooling (Barendregt et al., 2013) to stabilize variances, with results back-transformed for interpretation. We also provide a clearer description of heterogeneity assessment using Cochran’s Q and I² statistics with predefined thresholds. In addition, we have strengthened the description of our approach to handling heterogeneity and publication bias to improve transparency and methodological rigor.

#2. Publication bias assessment using funnel plots and Egger’s test may not be appropriate for prevalence meta-analysis. The manuscript uses funnel plots and Egger’s test. - For prevalence studies with substantial heterogeneity (I² = 99.64% as reported in the study) and proportional data, these methods are very unreliable. Please consider using Doi plots and the LFK index, which are more suitable for prevalence meta-analyses.

Response: Thank you, dear reviewer. We have now conducted a Doi plot with LFK index as recommended for prevalence meta-analyses. The LFK index was -0.27, indicating no significant small-study effects. The Doi plot now appears as Figure 4 in the main manuscript. In the revised manuscript, we report the LFK index value and interpret symmetry accordingly. The funnel plot has been removed from the main results.

#3. The quality assessment section contains inconsistencies regarding the appraisal tool used. The manuscript refers to the “JBI checklist for observational studies,” but the cited reference corresponds to methodological guidance for prevalence studies. Please specify the exact name of the appraisal tool used (e.g., “Joanna Briggs Institute Critical Appraisal Checklist for Studies Reporting Prevalence Data”) and provide the appropriate citation.

Response: Thank you for your insightful comment. We have corrected this. We now cite the Joanna Briggs Institute Critical Appraisal Checklist for Studies Reporting Prevalence Data specifically, with the appropriate reference. We now cite the checklist correctly and provide the full reference. The checklist itself is included as Supplementary File S3.

#4. The method used to categorize study quality into low, medium, and high quality appears arbitrary and should be justified. The manuscript categorizes studies using cut-offs of 0–4, 5–7, and 8–9. Please cite appropriate reference and also please clarify whether these thresholds were pre-specified in the protocol or developed post hoc. If they were not protocol-defined, this should be acknowledged as a methodological deviation.

Response: We thank the reviewer for this important observation. We would like to clarify that the study protocol did not pre-specify any JBI score thresholds for categorizing studies into low-, medium-, or high-quality groups (0–4, 5–7, and 8–9). The protocol specified only that the Joanna Briggs Institute (JBI) Critical Appraisal Checklist for Studies Reporting Prevalence Data would be used to assess methodological quality and risk of bias. We acknowledge that the quality categories (0–4, 5–7, and 8–9) were derived post hoc from the 0–9 JBI scoring range and were used solely for subgroup analysis. We also identified an error in the original manuscript stating that “only studies scoring ≥5 (medium or high quality) were included.” This statement was incorrect. No studies were excluded based on their JBI quality scores, and all eligible studies were retained in the review regardless of their appraisal results. In fact, all included studies scored between 7 and 9 on the JBI checklist (21 studies scored 8–9, and 4 studies scored 7) (Supplementary file 3). We have revised the manuscript by removing the low-, medium-, and high-quality classifications, as well as the statement indicating that studies scoring ≥5 were included. We now clarify that JBI appraisal was conducted to assess methodological quality and risk of bias.

#5. The approach used for resolving disagreements in quality assessment may not be methodologically appropriate. The manuscript states that discrepancies were resolved “by averaging the scores.” Typically, disagreements in critical appraisal are resolved through discussion or involvement of a third reviewer rather than averaging numerical scores. Please justify this approach or revise accordingly.

Response: We thank the reviewer for this important observation and agree that averaging quality assessment scores is not the preferred approach for resolving discrepancies in critical appraisal. We have therefore revised the manuscript accordingly. In the revised manuscript, disagreements between reviewers during the quality assessment process were resolved through discussion and consensus. Where consensus could not be reached, a third reviewer (senior author) made the final decision. The statement indicating that discrepancies were resolved by averaging scores has been removed.

#6. The rationale for pooling highly heterogeneous institution-based studies across SSA requires stronger justification. All included studies were institution-based cross-sectional studies from varying settings and healthcare systems. Pooling such heterogeneous data may limit interpretability and generalizability. The manuscript should better justify the appropriateness of pooled regional estimates and discuss contextual variability more explicitly.

Response: We thank the reviewer for this important observation. We agree that substantial clinical and methodological heterogeneity exists across institution-based studies conducted in different healthcare settings within sub-Saharan Africa. However, all included studies applied the Robson Ten-Group Classification System, which provides a standardized and internationally recognized framework for assessing cesarean section practices. This common classification enabled the synthesis of primary cesarean section rates across studies despite differences in healthcare systems and facility characteristics. To address this concern, we have strengthened the justification for pooling in the manuscript. Specifically, we now explain that a random-effects model was used to account for between-study variability and that the pooled estimate should be interpreted as a regional facility-based summary rather than a population-level prevalence estimate. We have also expanded the Discussion to more explicitly acknowledge contextual variability across settings, including differences in referral patterns, facility level, case mix, healthcare resources, and clinical practices. In addition, we conducted univariable meta-regression analyses using study period, geographic region, country income category, institution type, facility scope, and cesarean section cost-exemption policy to further explore sources of heterogeneity. None of these study-level characteristics significantly explained the observed between-study variability, suggesting that heterogeneity is likely driven by multiple interacting clinical and health-system factors not captured in the available data.

#7. The manuscript should clarify whether the pooled estimate represents population-level or facility-level cesarean section patterns. Since all included studies are institution-based and many appear tertiary/referral-center based, the pooled estimate likely reflects facility-level patterns rather than regional population prevalence. This distinction should be emphasized throughout the manuscript, particularly in the abstract, discussion, and conclusions.

Response: Thank you. We have revised the manuscript throughout to clearly state that the pooled primary cesarean section rate reflects facility-based births only. This is now emphasized in the abstract, methods, results, discussion, and conclusion. A new sentence in the limitations explicitly states that the estimates do not represent population-level prevalence due to underrepresentation of home births and primary-level facilities.

#8. Some interpretations regarding “overuse” of cesarean section appear overly assertive given the nature of the included data. The manuscript repeatedly interprets elevated PCS rates as evidence of “overuse.”

However, many included studies are from tertiary referral hospitals managing high-risk obstetric populations. Without detailed indication-level data, caution is needed when attributing findings to inappropriate or unnecessary cesarean use.

Response: We agree with the reviewer. We have systematically softened language throughout the manuscript, replacing “overuse” with “potential overuse,” “possible overuse,” “may indicate overuse,” or “raises concern for overuse.” We also added explicit cautions that without detailed indication data, definitive attribution to overuse is not possible. Reviewer #1 also raised a related point about causal language (CS → mortality), which we have addressed.

Additional editorial changes:

• All figures have been updated where indicated.

• The data availability statement remains correct and the Zenodo repository is active.

• The reference list has been checked for consistency.

We believe the manuscript is now methodologically sound and appropriately cautious in its interpretations. We respectfully request that the manuscript be accepted for publication.

Sincerely,

Kindu Yinges Wondie (corresponding author)

On behalf of all co-authors

Attachment Submitted filename: Response_to_Reviewers_auresp_2.docx

# Peer review: Decision Letter 2

**DOI:** 10.1371/journal.pone.0354911.r005

**Authors:** Pijush Kanti Khan

14 Jul 2026

Primary cesarean section in sub-Saharan Africa: a systematic review and meta-analysis using the Robson Ten-Group Classification System

PONE-D-25-55783R2

Dear Dr. Wondie,

We’re pleased to inform you that your manuscript has been judged scientifically suitable for publication and will be formally accepted for publication once it meets all outstanding technical requirements.

Within one week, you’ll receive an e-mail detailing the required amendments. When these have been addressed, you’ll receive a formal acceptance letter and your manuscript will be scheduled for publication.

An invoice will be generated when your article is formally accepted. Please note, if your institution has a publishing partnership with PLOS and your article meets the relevant criteria, all or part of your publication costs will be covered. Please make sure your user information is up-to-date by logging into Editorial Manager at Editorial Manager® and clicking the ‘Update My Information' link at the top of the page. For questions related to billing, please contact billing support.

If your institution or institutions have a press office, please notify them about your upcoming paper to help maximize its impact. If they’ll be preparing press materials, please inform our press team as soon as possible -- no later than 48 hours after receiving the formal acceptance. Your manuscript will remain under strict press embargo until 2 pm Eastern Time on the date of publication. For more information, please contact onepress@plos.org.

Kind regards,

Pijush Kanti Khan, Ph.D.

Academic Editor

PLOS One

Additional Editor Comments (optional):

Reviewers' comments:

Reviewer's Responses to Questions

**Comments to the Author**

1. If the authors have adequately addressed your comments raised in a previous round of review and you feel that this manuscript is now acceptable for publication, you may indicate that here to bypass the “Comments to the Author” section, enter your conflict of interest statement in the “Confidential to Editor” section, and submit your "Accept" recommendation.

Reviewer #2: All comments have been addressed

**********

2. Is the manuscript technically sound, and do the data support the conclusions?

The manuscript must describe a technically sound piece of scientific research with data that supports the conclusions. Experiments must have been conducted rigorously, with appropriate controls, replication, and sample sizes. The conclusions must be drawn appropriately based on the data presented.

Reviewer #2: Yes

**********

3. Has the statistical analysis been performed appropriately and rigorously?

Reviewer #2: Yes

**********

4. Have the authors made all data underlying the findings in their manuscript fully available?

The PLOS Data policy requires authors to make all data underlying the findings described in their manuscript fully available without restriction, with rare exception (please refer to the Data Availability Statement in the manuscript PDF file). The data should be provided as part of the manuscript or its supporting information, or deposited to a public repository. For example, in addition to summary statistics, the data points behind means, medians and variance measures should be available. If there are restrictions on publicly sharing data—e.g. participant privacy or use of data from a third party—those must be specified.

Reviewer #2: Yes

**********

5. Is the manuscript presented in an intelligible fashion and written in standard English?

PLOS ONE does not copyedit accepted manuscripts, so the language in submitted articles must be clear, correct, and unambiguous. Any typographical or grammatical errors should be corrected at revision, so please note any specific errors here.

Reviewer #2: Yes

**********

6. Review Comments to the Author

Please use the space provided to explain your answers to the questions above. You may also include additional comments for the author, including concerns about dual publication, research ethics, or publication ethics. (Please upload your review as an attachment if it exceeds 20,000 characters)

Reviewer #2: The authors have adequately addressed all of the comments and concerns raised during the review process. The revisions have significantly improved the quality, clarity, and overall presentation of the manuscript. The responses provided are satisfactory, and the modifications made are appropriate and well justified. In its current form, the manuscript appears to meet the required standards for publication.

**********

7. PLOS authors have the option to publish the peer review history of their article (what does this mean?). If published, this will include your full peer review and any attached files.

If you choose “no”, your identity will remain anonymous but your review may still be made public.

**Do you want your identity to be public for this peer review?**  For information about this choice, including consent withdrawal, please see our Privacy Policy.

Reviewer #2: **Yes:** Dr Anuj Kumar Pandey

**********

# Editor report: Acceptance letter

**DOI:** 10.1371/journal.pone.0354911.r006

**Authors:** Pijush Kanti Khan

PONE-D-25-55783R2

PLOS One

Dear Dr. Wondie,

I'm pleased to inform you that your manuscript has been deemed suitable for publication in PLOS One. Congratulations! Your manuscript is now being handed over to our production team.

At this stage, our production department will prepare your paper for publication. This includes ensuring the following:

* All references, tables, and figures are properly cited

* All relevant supporting information is included in the manuscript submission,

* There are no issues that prevent the paper from being properly typeset

You will receive further instructions from the production team, including instructions on how to review your proof when it is ready. Please keep in mind that we are working through a large volume of accepted articles, so please give us a few days to review your paper and let you know the next and final steps.

Lastly, if your institution or institutions have a press office, please let them know about your upcoming paper now to help maximize its impact. If they'll be preparing press materials, please inform our press team within the next 48 hours. Your manuscript will remain under strict press embargo until 2 pm Eastern Time on the date of publication. For more information, please contact onepress@plos.org.

You will receive an invoice from PLOS for your publication fee after your manuscript has reached the completed accept phase. If you receive an email requesting payment before acceptance or for any other service, this may be a phishing scheme. Learn how to identify phishing emails and protect your accounts at https://explore.plos.org/phishing.

If we can help with anything else, please email us at customercare@plos.org.

Thank you for submitting your work to PLOS ONE and supporting open access.

Kind regards,

PLOS ONE Editorial Office Staff

on behalf of

Dr. Pijush Kanti Khan

Academic Editor

PLOS One
